# A Novel In Vivo Model of Laryngeal Papillomavirus-Associated Disease Using *Mus musculus* Papillomavirus

**DOI:** 10.3390/v14051000

**Published:** 2022-05-08

**Authors:** Renee E. King, Andrea Bilger, Josef Rademacher, Ella T. Ward-Shaw, Rong Hu, Paul F. Lambert, Susan L. Thibeault

**Affiliations:** 1 McArdle Laboratory for Cancer Research, Department of Oncology, University of Wisconsin-Madison, Madison, WI 53705, USA; renee.king@wisc.edu (R.E.K.); bilger@oncology.wisc.edu (A.B.); etward@wisc.edu (E.T.W.-S.); plambert@wisc.edu (P.F.L.); 2 Department of Surgery, University of Wisconsin-Madison, Madison, WI 53705, USA; rademacher3@wisc.edu; 3 Department of Pathology and Laboratory Medicine, University of Wisconsin-Madison, Madison, WI 53705, USA; rhu6@wisc.edu

**Keywords:** mouse, papillomavirus, larynx, vocal folds, recurrent respiratory papillomatosis, RRP, laryngoscopy, MmuPV1

## Abstract

Recurrent respiratory papillomatosis (RRP), caused by laryngeal infection with low-risk human papillomaviruses, has devastating effects on vocal communication and quality of life. Factors in RRP onset, other than viral presence in the airway, are poorly understood. RRP research has been stalled by limited preclinical models. The only known papillomavirus able to infect laboratory mice, *Mus musculus* papillomavirus (MmuPV1), induces disease in a variety of tissues. We hypothesized that MmuPV1 could infect the larynx as a foundation for a preclinical model of RRP. We further hypothesized that epithelial injury would enhance the ability of MmuPV1 to cause laryngeal disease, because injury is a potential factor in RRP and promotes MmuPV1 infection in other tissues. In this report, we infected larynges of NOD scid gamma mice with MmuPV1 with and without vocal fold abrasion and measured infection and disease pathogenesis over 12 weeks. Laryngeal disease incidence and severity increased earlier in mice that underwent injury in addition to infection. However, laryngeal disease emerged in all infected mice by week 12, with or without injury. Secondary laryngeal infections and disease arose in nude mice after MmuPV1 skin infections, confirming that experimentally induced injury is dispensable for laryngeal MmuPV1 infection and disease in immunocompromised mice. Unlike RRP, lesions were relatively flat dysplasias and they could progress to cancer. Similar to RRP, MmuPV1 transcript was detected in all laryngeal disease and in clinically normal larynges. MmuPV1 capsid protein was largely absent from the larynx, but productive infection arose in a case of squamous metaplasia at the level of the cricoid cartilage. Similar to RRP, disease spread beyond the larynx to the trachea and bronchi. This first report of laryngeal MmuPV1 infection provides a foundation for a preclinical model of RRP.

## 1. Introduction

Recurrent respiratory papillomatosis (RRP) is an aggressive upper airway disease characterized by rapidly growing benign epithelial lesions that result from infection with human papillomavirus (HPV) [1,2,3]. Around 95% of RRP lesions are HPV positive, and around 85% are positive for low-risk mucosal types 6 and/or 11 alone [4]. The larynx and specifically the vocal folds are most often affected [5]. Since the vocal fold epithelium is essential for optimal voice production [6], the most common clinical symptom of RRP is impaired vocal quality and function [2,7]. This results in significant communication impairment and decreased quality of life in patients with RRP [8,9,10]. The morbidity of RRP is exceedingly high due to the recurrent nature of the disease and a lack of satisfactory treatment options. RRP symptom management is limited to repeated surgical procedures to remove lesions [11,12]. Procedures are uncomfortable, costly, and inconvenient for patients, and can injure and scar vocal fold tissues, which further impairs voice production [11]. Although emerging adjuvant medical treatments increase intersurgical intervals in some patients, surgery remains the standard of care [13,14,15,16]. Prophylactic vaccination with Gardasil 9 or Gardasil is protective against HPV 6 and 11, and juvenile-onset RRP incidence has significantly decreased in countries where vaccination rates are high [17,18,19]. However, there is high vaccine hesitancy in some high-income countries, including the United States, and disparate global access to vaccines [13]. In addition, most evidence indicates that HPV vaccination cannot prevent infection or disease in people who are already infected [20,21]. Therefore, vaccination may prevent RRP in the distant future, but in the meantime, there remains an urgent, unmet need for research into the prevention and treatment of RRP.

The incidence of RRP has been estimated at roughly 4 in 100,000 children and 2 in 100,000 adults in the United States [13]. Disease onset in children is associated with the presence of maternal genital warts during pregnancy or delivery, vaginal delivery, and firstborn children [22,23,24]. Disease onset in adults is associated with increased lifetime number of sexual partners [25]. These risk factors largely suggest modes of HPV transmission: vertically, in the case of pediatric patients, and sexually, in the case of adult patients. HPV DNA can be found in the upper respiratory mucosa of 20–30% of healthy school-aged children [26,27]. This prevalence of infection is 3–4 orders of magnitude higher than the incidence of juvenile-onset RRP. This discrepancy indicates that factors other than viral presence in the airway are required for the virus to establish infections that lead to RRP disease. One potential factor is epithelial injury. Papillomavirus infection begins in the proliferating basal cells of stratified squamous epithelia, which the virion is thought to access via a microwound [28]. Vocal fold mucosa oscillates hundreds of times per second during voice production [29,30], which poses a continual mechanical challenge to the laryngeal epithelium [31]. Excessively loud voice use can quickly compromise vocal fold epithelial barrier integrity [32,33,34]. Vocal folds can be injured during laryngeal surgical procedures or intubation for numerous medical indications [31]. Due to the position of the larynx between the respiratory and gastrointestinal tracts, the vocal folds are vulnerable to environmental exposures, including pollution, cigarette smoke, and gastric reflux [31,35]. Chronic exposure to these chemicals increases the permeability of the vocal fold epithelium over time [31] and, therefore, potentially viral access to basal epithelial cells. Clinical evidence regarding the role of vocal fold epithelial injury in RRP is limited to scant observational studies. Laryngopharyngeal reflux may be more common in patients with RRP [36], but a causal relationship has not been established. Evidence regarding smoking is equivocal [25,37]. Other chemical exposures and mechanical factors, such as intubation, laryngeal surgery, and voice production, have not yet been studied clinically in regard to their influence on RRP. 

An animal model of laryngeal papillomavirus infection and disease would facilitate experimental studies of the role of injury in RRP. In the long term, an animal model would also be ideal to model long-term infection and disease pathogenesis, latency, and recurrence—characteristic features of RRP [38,39,40]. Models of low-risk HPV-associated diseases, including RRP, are limited [41]. Current preclinical models of RRP are limited to in vitro organotypic raft culture and xenografts, which are typically used at early passages to study short-term viral and host gene expression [42,43,44,45]. In the sole reported experimental infection of an animal larynx with an animal papillomavirus, canine oral papillomavirus (COPV) was injected into the supraglottic laryngeal tissues of canines [46]. No macro- or microscopic disease appeared at any laryngeal injection sites, but some of the animals developed epiglottic lesions, which authors attributed to incidental intraoperative abrasion that allowed the virus to access the basal epithelium [46]. Vocal fold infection was not attempted and no follow-up work has been published. 

Species-specific tropism is a hallmark of papillomaviruses. A papillomavirus capable of infecting and causing disease in laboratory mice was not known until a 2011 report that identified what is now called *Mus musculus* papillomavirus (MmuPV1) [47]. MmuPV1 can infect both cutaneous and mucosal epithelium and cause benign papilloma and cancer in many tissues [48,49,50]. MmuPV1 has been used to create novel preclinical models of HPV-associated mucosal disease in the cervix [51], anus [52], and head and neck [53,54,55]. Epithelial injury is typically performed in experimental MmuPV1 infection models, particularly in the skin [56]. However, experimental wounding is not strictly necessary for MmuPV1 infection in mucosa. MmuPV1 infection can spread spontaneously throughout the genital, anal, and oral mucosa of immunocompromised mice [57], can arise in genital tissues that are experimentally infected but not wounded [58], and can be sexually transmitted by females to males and by males to females without experimental manipulation [59]. It is possible that mucosal tissues are naturally susceptible to MmuPV1, but the role of epithelial abrasion induced by grooming, chewing and swallowing hard chow, or sexual intercourse at these sites cannot be ruled out. Given the broad tissue tropism of MmuPV1, we hypothesized that this virus would readily infect the mouse larynx. Incidental injuries within a mouse larynx are highly unlikely. Due to aseptic housing and the predominance of ultrasonic vocalization, which does not involve vocal fold vibration [60,61], murine vocal folds are likely met with very few environmental or mechanical challenges to the integrity of their laryngeal epithelium. Thus, a mouse model is ideal to test the effect of epithelial injury on laryngeal papillomavirus disease in the short term, and to provide a foundation for our long-term goal to develop a preclinical model of RRP in a genetically tractable laboratory animal. 

In this paper, we report a novel model of laryngeal papillomavirus disease induced by MmuPV1. We leveraged technical advances in murine oropharyngeal procedures under gas anesthesia [55] and endoscopic laryngeal procedures [62,63] to infect the mouse larynx with MmuPV1. We found that MmuPV1 infected the larynx and vocal folds of immunocompromised mice and caused disease as early as 1 week post infection. Allowing disease to progress for 3 months resulted in severe dysplasia and cancer. Laryngeal lesions were positive for MmuPV1 transcripts, but negative for capsid protein, except for one case of squamous metaplasia. Our companion article (forthcoming) characterizes laryngeal epithelial changes induced by MmuPV1-associated disease. Disease incidence and severity were higher at earlier timepoints in mice that underwent vocal fold abrasion and infection than in those that were infected without abrasion, indicating that injury enhanced the ability of MmuPV1 to cause laryngeal disease. However, primary laryngeal infection resulted in laryngeal disease in all mice by 3 months, with or without vocal fold abrasion. In addition, we found that secondary laryngeal infections and disease arose by 5 months in mice that had been experimentally infected with MmuPV1 on their skin, and that had not undergone any experimental laryngeal or oral manipulations. Our findings demonstrate that injury is dispensable for laryngeal infection and disease in immunocompromised mice, while our methods provide a foundation for a small animal model of RRP. 

## 2. Materials and Methods

### 2.1. Study Design

**Figure 1:**
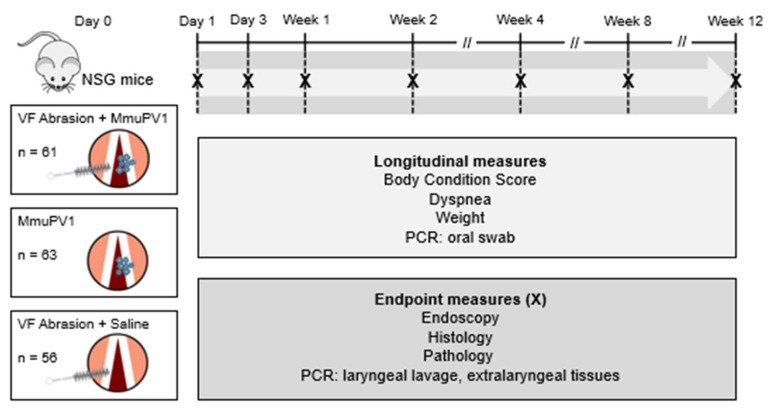
Study design.

Mice underwent 1 of 3 treatments: (1) vocal fold abrasion and MmuPV1 infection, (2) infection only, or (3) abrasion and mock infection. Two cages (1 per sex) of 3–5 mice each were sacrificed on days 1 and 3 and weeks 1, 2, 4, 8, and 12 post treatment. In addition, 2 cages (1 per sex) underwent mock infection without abrasion and were sacrificed at week 12, and 2 cages served as naïve controls. Data and samples were collected weekly and at endpoints as depicted in Figure 1. We compared measures of laryngeal MmuPV1 infection and disease pathogenesis between mice that underwent vocal fold abrasion and mice whose larynges were uninjured. Mice that underwent vocal fold abrasion and mock infection were included to control for the effect of wound healing on health outcomes and tissue pathology.

### 2.2. Animals

Equal numbers of adult male and female NOD scid gamma (NSG) mice [64] aged 5–10 weeks of age were used for this study. A total of 193 NSG mice were used, comprising 3–5 mice per sex per treatment group per experimental endpoint. Mice were purchased from Jackson Laboratory (stock #005557; Bar Harbor, ME, USA) and bred by the University of Wisconsin Biomedical Research Model Services Laboratory. Mice were housed in aseptic conditions in the University of Wisconsin School of Medicine and Public Health Animal Care Unit, which is accredited by the Association for Assessment and Accreditation of Laboratory Animal Care. Animal procedures were approved by the University of Wisconsin-Madison Institutional Animal Care and Use Committee (IACUC #M005871) and conducted in accordance with the National Institutes of Health (NIH) *Guide for the Care and Use of Laboratory Animals* [65]. More male than female humans are diagnosed with adult onset RRP [25,66]. To monitor differences in susceptibility of male versus female mice to MmuPV1-induced laryngeal disease, we balanced sexes equally in our experimental cohorts. 

### 2.3. Laryngoscopy

**Figure 2:**
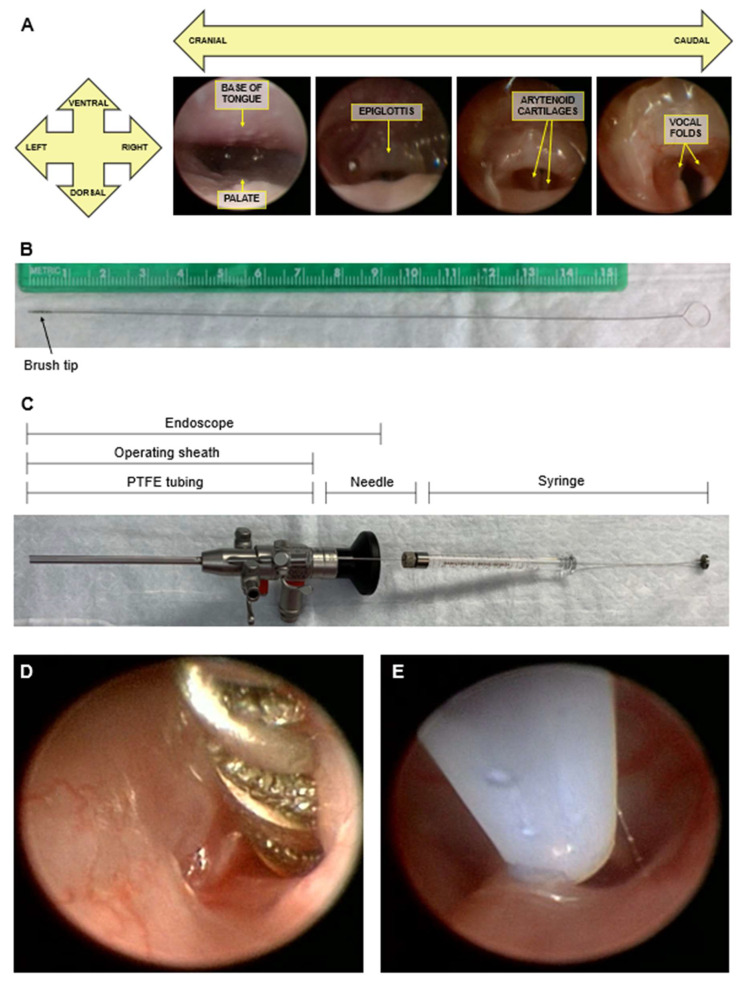
Murine endoscopic laryngeal procedures. (**A**) Endoscopic images of pharynx and larynx with anatomic orientation and relevant tissues labeled, progressing from the back of the oral cavity (cranial) to the larynx (caudal). (**B**) Custom stainless steel micro brush for vocal fold abrasion with 0.5 mm diameter. (**C**) Gastight 10 uL syringe, 26-gauge needle, and 26-gauge PTFE tubing within the assembled operating sheath and endoscope. (**D**) Endoscopic image of murine vocal fold abrasion. Brush bristles are apparent between spiral wire. (**E**) Endoscopic image of murine vocal fold inoculation. Drops of inoculate are visible within tubing. Clear tubing appears white due to image magnification and reflected light.

Mice underwent videolaryngoscopy for vocal fold abrasion and/or laryngeal inoculation (Figure 2). All procedures were completed inside a biosafety cabinet. Anesthesia was induced with 5% isoflurane and maintained at 2% throughout laryngeal procedures. Endoscopic access to the oropharyngeal cavity under gas anesthesia was facilitated by the use of a custom-designed nose cone as previously described [55]. The nose cone exploits rodents’ status as obligate nasal breathers to continuously administer isoflurane gas during oral manipulations, allowing oral and pharyngeal procedures without injected anesthetics. Maxillary incisors and mandible were secured within the nose cone by silicone O-rings, and a large window in the nose cone allowed access to the oral cavity [55]. Access to the larynx was achieved by placing an additional O-ring in an X-shaped sling within the nose cone to raise the head and extend the neck. 

Tissues were visualized using a 1.9 mm diameter 30° Hopkins endoscope (64301 BA, Karl Storz, El Segundo, CA, USA) within a 2.5 mm diameter operating sheath with a 3 French (~1 mm) channel (61029 D, Karl Storz, El Segundo, CA, USA), xenon light source (Kay Elemetrics Xenon 7150, KayPENTAX, Inc., Lincoln Park, NJ, USA), and lightweight color camera (Tricam NTSC Camera System, Karl Storz, El Segundo, CA, USA). Endoscopic images were recorded on a high-definition video capture system (SDC HD, Stryker Corporation, Kalamazoo, MI, USA). Videos were played back frame by frame in Adobe Premiere Pro (Adobe Inc., San Jose, CA, USA) for analysis and screen capture.

### 2.4. Vocal Fold Abrasion

Vocal fold epithelium was abraded using a 0.5 mm diameter stainless steel miniature single-spiral brush with bristles (Figure 2B; custom fabrication, Gordon Brush Mfg. Co. Inc., City of Industry, CA, USA). The brush was advanced through the channel of the operating sheath into the larynx and rubbed against the vocal fold epithelium 5–10 times (Figure 2D). 

### 2.5. MmuPV1 Infection

High-titer crude stocks of MmuPV1 were prepared from homogenized MmuPV1-induced warts as previously described [55]. The concentration of virus stock used for these experiments was 2.6 × 10^9^ viral genome equivalents (VGE) per ul. All experiments were completed with virus from the same prep.

Under laryngoscopy, the larynx was inoculated topically with 2 uL of either MmuPV1 virus stock (total VGE 5.2 × 10^9^) or sterile phosphate-buffered saline (PBS, Fisher Scientific, Hampton, NH, USA). Inoculate was delivered using medical grade polytetrafluroethylene (PTFE) tubing (BB311-32, Scientific Commodities, Inc., Lake Havasu City, AZ, USA; 26 gauge, 1 mm outer diameter) attached to a gastight syringe (1700 series, 7653-01, Hamilton Company, Reno, NV, USA; 10 ul) with a removable needle (7804-03, Hamilton Company, Reno, NV, USA; 26 gauge, 51 mm). Tubing was cut to the length of the operating sheath and fed through the sheath’s working channel (Figure 2C). This system is airtight from the syringe to the tip of the tubing, allowing solutions to be drawn up and deposited similar to using a pipette. Precise measurement is facilitated by the hubless design of the syringe, which ensures that, when the plunger is withdrawn to the 2 uL mark, 2 uL of liquid is drawn up at the tip of the tube. This was verified in preliminary experiments (data not shown). Inoculate was drawn up into the tip of the tube, the tube was advanced through the channel of the operating sheath until it reached into the larynx, and the larynx was inoculated (Figure 2E). New tubing was used for each animal. Infection or mock infection was achieved immediately after injury for the mice that underwent vocal fold abrasion.

### 2.6. Animal Health

Health of all mice was assessed weekly. Specific measures included Body Condition Score (BCS) [67], body weight (g), and the presence or absence of dyspnea and stridor. BCS was performed by palpating the iliac crest and spinal column and rating body condition on a 5-point scale, where 5 = obesity resulting in inability to feel bones, 3 = optimal condition, and 1 = emaciated [67,68]. It was determined a priori per IACUC protocol that mice would be euthanized at BCS ≤ 2 or weight loss of 20%. Dyspnea was rated present if mice displayed shallow rapid breathing, irregular gasping, or abdominal effort. Stridor was rated present for audible high-pitched breathing.

### 2.7. Oral Swabs for Longitudinal MmuPV1 DNA Detection

Oral swabs for the polymerase chain reaction (PCR) detection of viral DNA were collected weekly. The swab procedure was modified from a previously described procedure for tracking oral MmuPV1 infection [53]. Sterile flocked swabs with a 3.4 mm diameter tip (PurFlock Ultra, 25-3318-U, Puritan Medical Products, Guilford, ME, USA) were placed dry into the oral cavity, rotated back and forth 10 times, withdrawn, placed in 200 uL sterile PBS, and stored at −20 °C. Samples were brought to room temperature before DNA isolation. Swab tubes were vortexed and the eluate was allowed to settle for 5 min before swabs were carefully removed from PBS and discarded. DNA was isolated as previously described [51] using spin columns (DNeasy Blood and Tissue kit, Qiagen, Hilden, Germany) following the manufacturer protocol for animal blood and cultured cells, and eluted in 50 uL elution buffer. MmuPV1 DNA was detected by PCR as previously described [51] using primers specific to the E2 gene (E2-1: 5′-GCC CGA AGA CAA CAC CGC CAC G-3′ and E2-2: 5′-CCT CCG CCT CGT CCC CAA ATG G-3′). The presence of adequate DNA in samples was verified by PCR for the *Mus musculus* p53 gene (primers p53-1: 5′-TAT ACT CAG AGC CGG CCT-3′ and p53-2: 5′-ACA GCG TGG TGG TAC CTT AT-3′). PCR products were analyzed by agarose gel electrophoresis. Controls were MmuPV1 virus stock or previously amplified and gel purified E2 gene (E2-positive, p53-negative) and uninfected larynx or tongue tissue (E2-negative, p53-positive).

### 2.8. Endoscopic Assessment of Lesions

After euthanasia, cadavers underwent videolaryngoscopy to assess papillomas throughout the larynx and pharynx. Images were obtained postmortem to avoid increased handling, anesthesia exposure, and risk of procedure complications in live animals. Endoscopic equipment and procedures were largely the same as described above, but the nose cone and operating sheath were unnecessary. Videos were recorded and assessed for grossly visible lesions or abnormalities at the vocal folds, epiglottis, arytenoids, aryepiglottic folds, palate, and base of tongue. Videos were played back frame by frame in Adobe Premiere Pro (Adobe Inc., San Jose, CA, USA) for analysis and screen capture. One reviewer assessed the videos, who was blinded to treatment group and timepoint post infection and/or injury.

### 2.9. Tissue Collection and Histology

Tissues collected included larynx, tongue, distal trachea and lungs, esophagus, and palate. Prior to fixation, laryngeal lavage was collected for DNA isolation and PCR by placing the dissected larynx into 200 uL sterile PBS in an Eppendorf tube and gently tapping the tube on a hard surface for 10 s. Lavage was stored at −20 °C. All larynges and tongues and half of the trachea–lung complexes and esophagi were fixed in 10% neutral buffered formalin (NBF) at room temperature for 24 h. The other half of the distal trachea, lung, and esophagus tissues were stored in cryotubes at −20 °C for DNA isolation and PCR. Palates were decalcified and fixed in Surgipath Decalcifier I (Leica Biosystems, Wetzlar, Germany) for a total of 48 h at room temperature, with a change to fresh decalcifier fixative after 24 h. Tissues were processed, embedded in paraffin, and sectioned into 5 um sections. Larynges were cut to obtain coronal sections. Larynx sections included the hypopharynx, which is separated from the oropharynx at the tip of the epiglottis in mice and humans [69]. Tongues and palates were sectioned sagittally. Trachea–lung complexes were sectioned longitudinally relative to the trachea. Esophagi were sectioned longitudinally. Every 10th slide was stained with hematoxylin and eosin (H&E).

### 2.10. Detection of MmuPV1 DNA in Larygneal Lavage, Extralaryngeal Tissue, and FFPE Slides

Lavage DNA was isolated using the DNeasy kit as described above for oral swabs. Tissue DNA was isolated using the DNeasy kit following the protocol for animal tissues and eluted in 400 uL elution buffer. DNA from select formalin-fixed, paraffin-embedded (FFPE) slides was isolated using the DNeasy kit following a procedure modified from manufacturer protocols and previously described methods [52]. Briefly, 4 tissue sections per sample (2 slides) were scraped into Eppendorf tubes using a fresh razor blade for each sample. Samples were deparaffinized with xylene for 5 min, centrifuged, and supernatant was removed, and deparaffinization was repeated. Samples were washed twice in 100% ethanol. The pellet was allowed to dry completely at room temperature, then resuspended in 180 uL tissue lysis buffer from the kit. DNA was isolated following the manufacturer protocol for animal tissue and eluted in 50 uL elution buffer. MmuPV1 DNA was detected by PCR as described above.

### 2.11. Pathology Grading

H&E-stained slides from all tissues were assessed by an experienced Head and Neck Pathologist blinded to treatment group and timepoint post treatment. Ratings included the presence of inflammation, papilloma, dysplasia, and cancer, and severity of any disease present. The severity of dysplasia was determined by the extent of abnormal cells above the basal layer of the epithelium. In this rating schema, severe dysplasia was considered the same as carcinoma in situ. Invasive cancer was defined as the invasion of epithelial cells beyond the basement membrane. Laryngeal subsites were scored separately, specifically vocal folds, epiglottis and ventral pouch, and arytenoids and aryepiglottic folds. Extralaryngeal tissues included in larynx slides were also scored separately, specifically proximal trachea and hypopharynx. 

### 2.12. RNAscope ISH for MmuPV1 RNA

In situ hybridization (ISH) for the MmuPV1 E4 transcript was completed for larynx slides from all mice with either pathologist-diagnosed laryngeal disease or MmuPV1 DNA detected with swab or lavage PCR. In infected groups without positive PCR or disease, larynx slides from 2 mice per sex per group per timepoint were stained. Larynx slides from all mice in the Abrasion + Saline group that were harvested at week 12 were stained to check for transcripts in mock-infected animals. Other tissues were stained as necessary. Slides were stained using RNAscope ISH (2.5 HD Reagent Kit-Brown, 322300, Advanced Cell Diagnostics, Newark, CA, USA) with probes specific for MmuPV1 E4 (473281) and L1 (473271) according to the manufacturer’s instructions. To distinguish viral transcript from viral DNA, select sections were incubated for 30 minutes prior to probe hybridization with either 20 U DNase I (EN0521, Fisher Scientific, Hampton, NH, USA) or 20 U DNase I, 2000 U RNase T1 (19101, Qiagen, Hilden, Germany), and 500 ug RNase A (EN0451, Fisher Scientific, Hampton, NH, USA) in 1X buffer containing MgCl_2_ [70]. To ensure specificity of MmuPV1 probes vs. background 3,3′-diaminobenzidine (DAB) staining, select sections were hybridized with a negative control probe provided by the manufacturer (310043). 

### 2.13. MmuPV1 L1 and K14 Dual Immunofluorescence

Larynx slides from all infected mice and from Abrasion + Saline mock-infected mice from week 12 were dual stained for MmuPV1 L1 capsid protein and cytokeratin 14 (K14). Other tissues were stained as necessary. Slides were stained using tyramide signal amplification (TSA) as previously described [71]. Primary antibodies were rabbit anti-L1 (1:5000; gift from Dr. Chris Buck, National Cancer Institute, National Institutes of Health, Bethesda, MD, USA) and rabbit anti-K14 (1:1000; 905301, Biolegend, San Diego, CA, USA). Secondary antibodies and chemicals to label L1 were goat anti-rabbit horseradish peroxidase (1:500; 111-035-144, Jackson ImmunoResearch Inc., West Grove, PA, USA), biotin-tyramide solution (1:500; stock 1 mg/ml, made with biotin N-hydroxysuccinimide ester (H1759, Sigma-Aldrich, St. Louis, MO, USA), tyramine hydrochloride (T2879, Sigma-Aldrich, St. Louis, MO, USA), triethylamine (25018, Fisher Scientific, Hampton, NH, USA), and N,N-dimethylformamide (227056, Sigma-Aldrich, St. Louis, MO, USA)), and streptavidin-conjugated Alexa Fluor 594 (1:500; S32356, Fisher Scientific, Hampton, NH, USA). Secondary antibody for K14 was goat anti-rabbit Alexa Fluor 488 (1:500; A-11008, Fisher Scientific, Hampton, NH, USA). Nuclei were counterstained with 1X Hoechst 33342 (H1399, Fisher Scientific, Hampton, NH, USA) for 10 min, then slides were mounted and coverslipped with Prolong Diamond mounting media (P36970, Fisher Scientific, Hampton, NH, USA), cured flat at room temperature in the dark for 24 h, and stored at 4 °C.

### 2.14. Image Acquisition

Images were acquired on a Nikon Eclipse Ti2 inverted microscope with NIS Elements software (Nikon, Tokyo, Japan). Tissues were photographed at 4× and 10× magnification, and the areas of interest were photographed at 40×.

### 2.15. Infectivity of Laryngeal Lavage

The infectivity of laryngeal lavage was tested in the skin of athymic nude mice (Foxn1^nu^). Six females were purchased from Envigo (Indianapolis, IN, USA) and used for experiments at 6–8 weeks of age. Six sites per animal, four on the tail and one on each ear, were scarified and inoculated. Two mice were mock-infected with PBS, two were infected with high-titer MmuPV1 virus stock, and two were infected with laryngeal lavage. Lavage was pooled from 12 NSG mice harvested on week 8 post laryngeal MmuPV1 infection. Nude mice were euthanized 5 months after skin infections. Ears, warts, skin, larynges, tongues, and palates were fixed, processed, embedded in paraffin, sectioned, and H&E stained as described above. Pathology was scored and select sections were stained for MmuPV1 E4 transcript, L1 capsid protein, and K14 as described above.

### 2.16. Statistical Analysis

Continuous variables were tested for normality using the Kolmogorov–Smirnov test. Group differences in age and weight at baseline were tested with the Kruskal–Wallis test. Group differences in sex were tested with the Fisher’s exact test. Weight data were converted to percentages of each animal’s baseline weight, and group differences in changes in weight over time were analyzed using type 3 fixed effects in linear mixed models. Post hoc pairwise differences in weight were tested using simple differences of least squares means with Tukey–Kramer adjustment. Differences in the presence of disease among head and neck tissues and between groups and sexes were tested with Fisher’s exact tests, with Bonferroni adjustment as necessary. Pathology ratings were converted to an ordinal scale where negative pathology = 0, mild dysplasia = 1, moderate dysplasia = 2, severe dysplasia = 3, and invasive cancer = 4. For some analyses, data from laryngeal tissues were pooled and the highest severity among vocal folds, epiglottis, or arytenoids was analyzed. Differences in severity of disease among head and neck tissues and between groups and sexes were tested with the Wilcoxon rank sum test or Kruskal–Wallis test with Dwass, Steel, Critchlow-Fligner (DSCF) pairwise post hoc test. Statistical analyses were conducted with SAS Studio 3.8 running SAS 9.4.1 (SAS Institute, Inc., Cary, NC, USA). Alpha level for significance was 0.05.

## 3. Results

### 3.1. Animal Health

Mice were studied for up to 12 weeks after laryngeal MmuPV1 infection and vocal fold abrasion, infection alone, or abrasion and mock infection (Figure 1). Of the 193 NSG mice used for this study, 187 (97%) survived to planned experimental endpoints, while 6 did not. Body condition was assessed as BCS = 3 (optimal condition) for almost all animals for the duration of the study. Only 6 mice (3%) had BCS < 3 at any point. Overall, there did not appear to be an association between treatment group and survival or BCS. Neither dyspnea nor stridor was observed in any animal throughout the study. Mixed models for weight change as a percentage of baseline weight revealed a significant Group × Time interaction, indicating that weight gain differed by treatment group (Table S1, Figure S1). Post hoc tests revealed that weight gain was 7–13% lower in infected groups at later timepoints and was lower in both infected groups than both mock-infected groups by week 12 (Figure S1, Table S2). There were no differences between groups of infected mice with and without vocal fold abrasion. Group × Time × Sex 3-way interaction was not significant and was removed from the model before interpretation. This indicates that changes in weight gain by infection and injury status did not differ between males and females. Taken together, these data indicate that NSG mice with laryngeal MmuPV1 infection remained in generally good health over 12 weeks, with a mild flattening of weight gain that did not affect body condition or survival. Vocal fold injury, alone or in addition to infection, did not affect animal health. 

### 3.2. MmuPV1 DNA in Oral Swabs and Laryngeal Lavage

Oral swab PCR for the MmuPV1 E2 gene revealed viral DNA in the oral cavity in some infected mice as early as week 1 and in most by week 2 post infection (Figure S2A). The percentage of virus-positive mice increased over time to 100% in infected groups weeks 6 and 7 (Figure S2). Mock-infected mice were negative for MmuPV1 DNA throughout. However, DNA quantity and quality were highly variable. The control host gene p53 was absent in most samples collected during weeks 9–12. For these reasons and because of the emergence of pathology throughout the head and neck region beyond the vocal folds, described below, oral swab PCR was determined to be a poor longitudinal measure of laryngeal-specific MmuPV1 infection and disease.

MmuPV1 DNA was present in laryngeal lavage as early as week 2 (Figure S3). Percent positive lavage samples increased from weeks 1–12. Similar to oral swab samples, the *Mus musculus* p53 gene was absent in many lavage samples, including all infected females collected at week 8. Thus, DNA quality and quantity often could not be verified. Based on the available data, there were no differences in viral DNA in laryngeal lavage by injury group or sex. Lavage from all mock-infected animals was negative for MmuPV1 DNA. 

### 3.3. Endoscopic Assessment of Lesions

**Figure 3:**
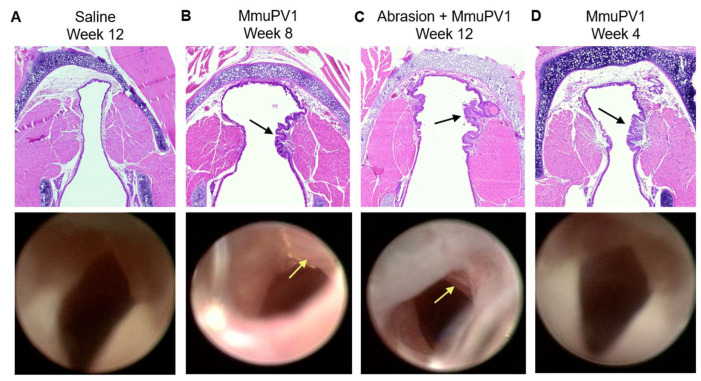
Gross and microscopic murine vocal fold lesions post MmuPV1 infection. H&E-stained histologic and endoscopic images of vocal folds from the same animal. (**A**) Week 12 post saline mock infection. (**B**) Week 8 post MmuPV1 infection. Arrows indicate grossly visible, smooth vocal fold lesion. (**C**) Week 12 post vocal fold abrasion and MmuPV1 infection. Arrows indicate grossly visible, smooth, pale vocal fold lesion. (**D**) Week 4 post MmuPV1 infection. Arrow indicates lesion on histology. Lesion was not visible on endoscopy.

Some vocal fold lesions were grossly visible on the postmortem endoscopy (Figure 3B,C). However, they were smooth, flat, and slightly paler than surrounding tissue, rather than the characteristic clustered, stippled appearance of RRP in human patients. Other vocal fold lesions revealed by histology were not visible in all endoscopic videos (Figure 3D). Thickened or bumpy epithelium was also observed on the laryngeal surface of the epiglottis in some animals (Figure S4). Vocal folds could not be visualized in all mice postmortem due to complications of euthanasia.

### 3.4. Pathology Grading

**Figure 4:**
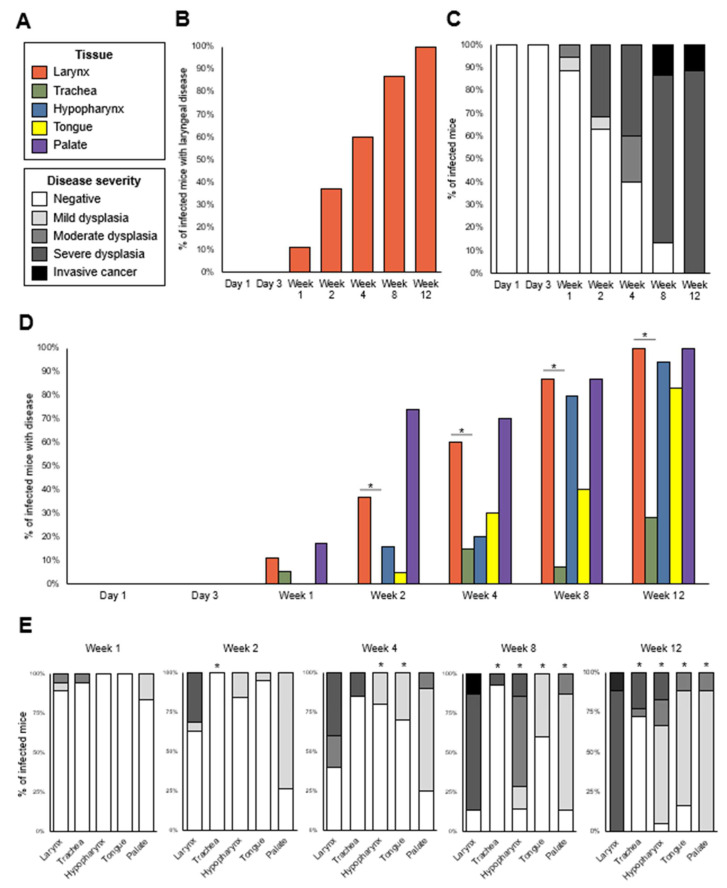
MmuPV1 induced severe laryngeal disease. Infected groups only, n = 15–20 mice per timepoint. (**A**) Key for panels (**B**)–(**E**). (**B**) Disease incidence in larynx for each animal. (**C**) Highest disease severity in larynx for each animal. (**D**) Disease incidence in larynx vs. other head and neck tissues. Fisher’s exact tests of differences in disease incidence among head/neck tissues at each timepoint: week 1, *p* = 0.2976; week 2, *p* < 0.0001; week 4, *p* = 0.0001; week 8, *p* < 0.0001; week 12, *p* < 0.0001. * Significant post hoc Bonferroni-adjusted Fisher’s exact pairwise tests between the larynx and other tissues: larynx vs. trachea week 2, *p* = 0.0080; week 4, *p* = 0.0079; week 8, *p* < 0.0001; week 12, *p* < 0.0001. All other pairwise tests vs. larynx: *p* > 0.05/4 = 0.0125. (**E**) Disease severity in larynx vs. other head and neck tissues. Severity was coded ordinally for analysis: negative = 0, mild dysplasia = 1, moderate dysplasia = 2, severe dysplasia = 3, invasive cancer = 4. Kruskal–Wallis tests of severity among tissues at each timepoint: week 1, *p* = 0.2480; week 2, *p* < 0.0001; week 4, *p* = 0.0001; week 8, *p* < 0.0001; week 12, *p* < 0.0001. * Significant post hoc DSCF test between larynx and other tissues: week 2 larynx vs. trachea *p* = 0.0322; week 4 larynx vs. hypopharynx *p* = 0.0121, larynx vs. tongue *p* = 0.0340; week 8 larynx vs. trachea *p* = 0.0003, larynx vs. hypopharynx *p* = 0.0140, larynx vs. tongue *p* = 0.0006, larynx vs. palate *p* = 0.0020; week 12 larynx vs. trachea *p* < 0.0001, larynx vs. hypopharynx *p* < 0.0001, larynx vs. tongue *p* < 0.0001, larynx vs. palate *p* < 0.0001. All other pairwise tests vs. larynx: adjusted *p* > 0.05.

Laryngeal epithelial disease was diagnosed as early as week 1 post infection, and in 100% of infected mice by week 12 (Figure 4B). Lesions were not papillomas, but dysplasias or invasive squamous cell carcinoma at all timepoints. Severe dysplasia emerged as early as week 2, while cancers were diagnosed by weeks 8 and 12 (Figure 4C). The injury of laryngeal tissues other than vocal folds could not be completely avoided in the abrasion procedure, and virus stock contacted the entire larynx during inoculation. Reflective of this, disease developed in the epiglottis/ventral pouch and arytenoid/aryepiglottic fold epithelium as well as vocal folds (Figure S5). Disease also emerged in the epithelium of extra-laryngeal head and neck tissues, including proximal trachea just caudal to the larynx, hypopharynx, tongue, and palate (Figure 4D). Due to the size of the mouse pharynx, it is likely that oral and pharyngeal tissues were exposed to MmuPV1 and possibly injured by the operating sheath during the transoral laryngeal infection procedure. Disease incidence in the larynx was higher than proximal trachea at weeks 2–12, but did not differ from hypopharynx, tongue, or palate (Figure 4D). However, the severity of laryngeal disease was higher than in all other head and neck tissues by weeks 8 and 12 (Figure 4E). This indicates that experimental procedures primarily targeted the larynx to induce MmuPV1 infection and disease.

**Figure 5:**
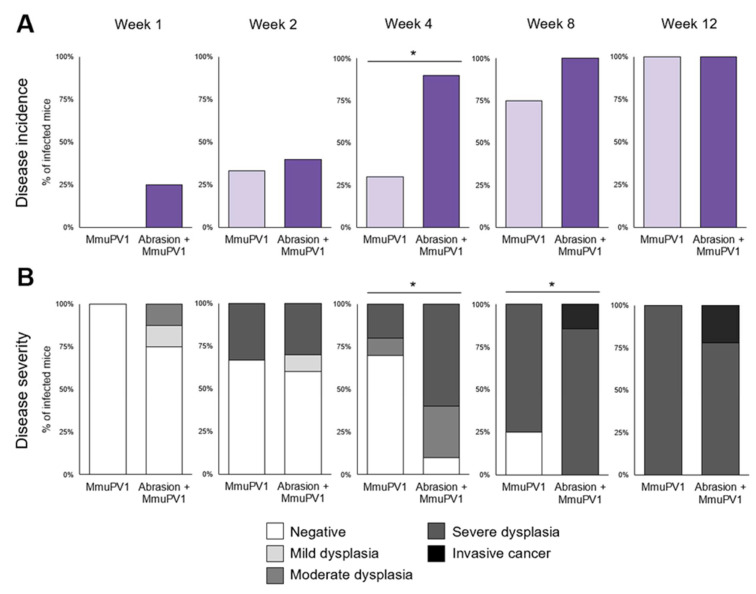
Injury enhanced, but was not necessary, for MmuPV1-induced laryngeal disease in NSG mice. Infected groups week 1 and later only, n = 7–10 mice per group per timepoint. (**A**) Laryngeal disease incidence after laryngeal MmuPV1 infection vs. vocal fold abrasion and infection. * Significant Fisher’s exact test of group difference in dysplasia incidence in the larynx: week 4, *p* = 0.0198. All other *p* > 0.05. (**B**) Laryngeal disease severity after laryngeal MmuPV1 infection vs. vocal fold abrasion and infection. Severity was coded ordinally: negative = 0, mild dysplasia = 1, moderate dysplasia = 2, severe dysplasia = 3, invasive cancer = 4. * One-tailed Wilcoxon rank-sum tests of severity in Abrasion + MmuPV1 vs. MmuPV1 at each timepoint: week 4, *p* = 0.0083; week 8, *p* = 0.0313. All other *p* > 0.05.

Abrasion was not necessary for MmuPV1-induced laryngeal dysplasia (Figure 5). All infected animals developed laryngeal disease by week 12, regardless of injury. However, incidence was higher in the Abrasion + MmuPV1 group than the MmuPV1 group at week 4 (Figure 5A), and severity was higher in the injured group at weeks 4 and 8 (Figure 5B). Thus, the abrasion enhanced disease pathogenesis in the MmuPV1-infected mouse larynx. There was no sex difference in laryngeal dysplasia incidence or severity (Figure S6).

Two mice that underwent vocal fold abrasion and mock infection were diagnosed with mild epiglottic dysplasia. These animals were negative for MmuPV1 DNA in oral swabs and laryngeal lavage, and tissues were negative for MmuPV1 transcript and protein in ISH and IF assays, as described below. To assess MmuPV1 DNA in tissues, sections were scraped from FFPE sections for 2 larynx slides per animal and DNA was isolated. PCR results revealed weak, but present, p53 bands, confirming the presence of DNA in the samples and the absence of MmuPV1 DNA (Figure S7). Overall, molecular assays indicate that rare laryngeal dysplasia in mock-infected mice was not associated with MmuPV1 infection. All control mice that underwent no laryngeal or oral procedures had negative pathology.

### 3.5. MmuPV1 RNA and Capsid Protein in Larynx

**Figure 6:**
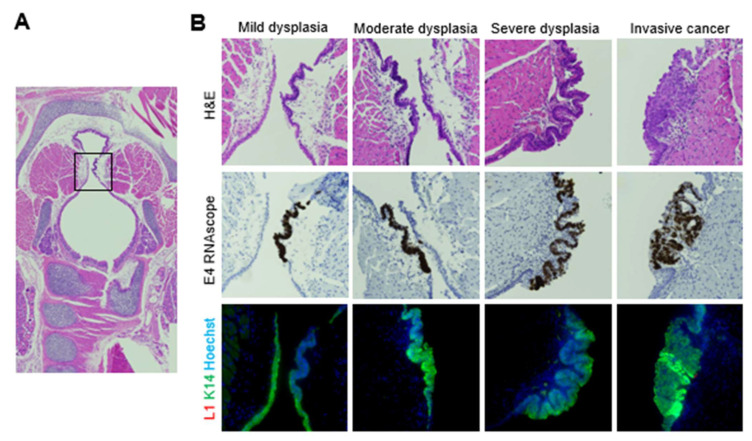
MmuPV1-induced vocal fold dysplasia was positive for E4 transcript and negative for L1 capsid protein, regardless of severity. (**A**) Low magnification of H&E-stained coronal section of murine larynx. Box indicates vocal folds. (**B**) Serial sections of mild, moderate, and severe vocal fold dysplasia stained with H&E, MmuPV1 E4 RNAscope ISH, and MmuPV1 L1/K14 IF. 40×.

Laryngeal disease was positive for MmuPV1 E4 transcript, but negative for L1 capsid protein, regardless of severity (Figure 6). RNAscope ISH for MmuPVL1 L1 transcript was completed in a subset of larynges. L1 transcripts colocalized with E4 transcripts (Figure S8). Thus, MmuPV1 transcription and disease occurred in the mouse larynx without productive infection, i.e., assembly of infectious virions. 

**Figure 7:**
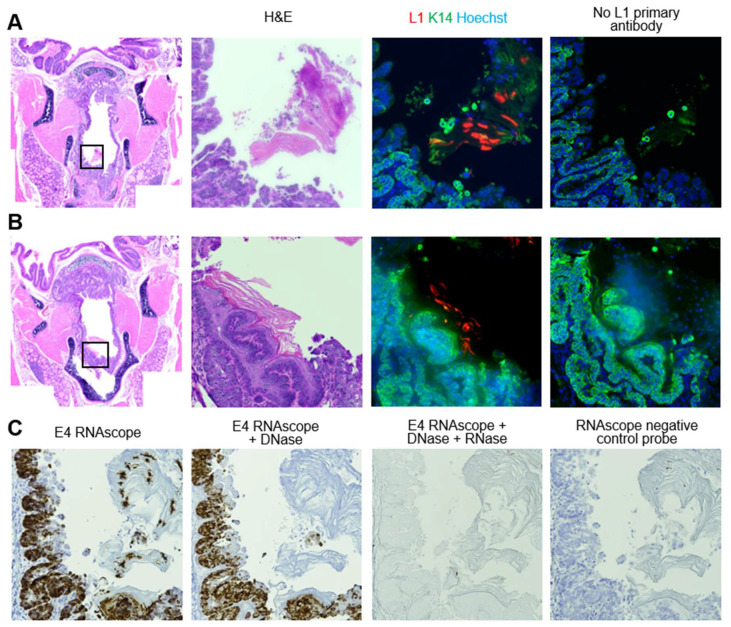
Squamous metaplasia and severe dysplasia at the level of the cricoid cartilage was positive for MmuPV1 transcripts and L1 capsid protein. (**A**) Low magnification of H&E-stained coronal section of murine larynx and 40× magnifications of area indicated in box and serial slides stained with H&E, MmuPV1 L1/K14 IF, and MmuPV1 L1/K14 IF with no L1 primary antibody. (**B**) Same stains as in (**A**), 10 slides (20 sections) more posterior within the larynx. (**C**) Sections serial to (**A**), stained with MmuPV1 E4 RNAscope ISH, E4 RNAscope with DNase treatment, E4 RNAscope with DNase and RNase treatment, and negative control probe RNAscope ISH.

Capsid protein was detected in the respiratory and/or intermediate epithelia of exactly one animal, at the level of the cricoid cartilage (Figure 7A,B). L1-positive epithelium was keratinized and desquamating from the epithelial surface. This is consistent with squamous metaplasia, a known response to injury in respiratory tissue [72]. RNAscope revealed viral transcripts in tissue and desquamating keratinized epithelium (Figure 7C). RNAscope is able to label both MmuPV1 RNA and DNA [70]. Signal persisted in tissue after DNase treatment, indicating that RNAscope primarily labeled transcripts. However, in desquamating epithelium, the signal was found in fewer foci after DNase treatment, suggesting that signal could represent amplified viral DNA potentially packaged into new virions. Negative control conditions for RNAscope consisting of DNase + RNase treatment and negative control probes revealed minimal background signal in the desquamating tissue. 

**Figure 8:**
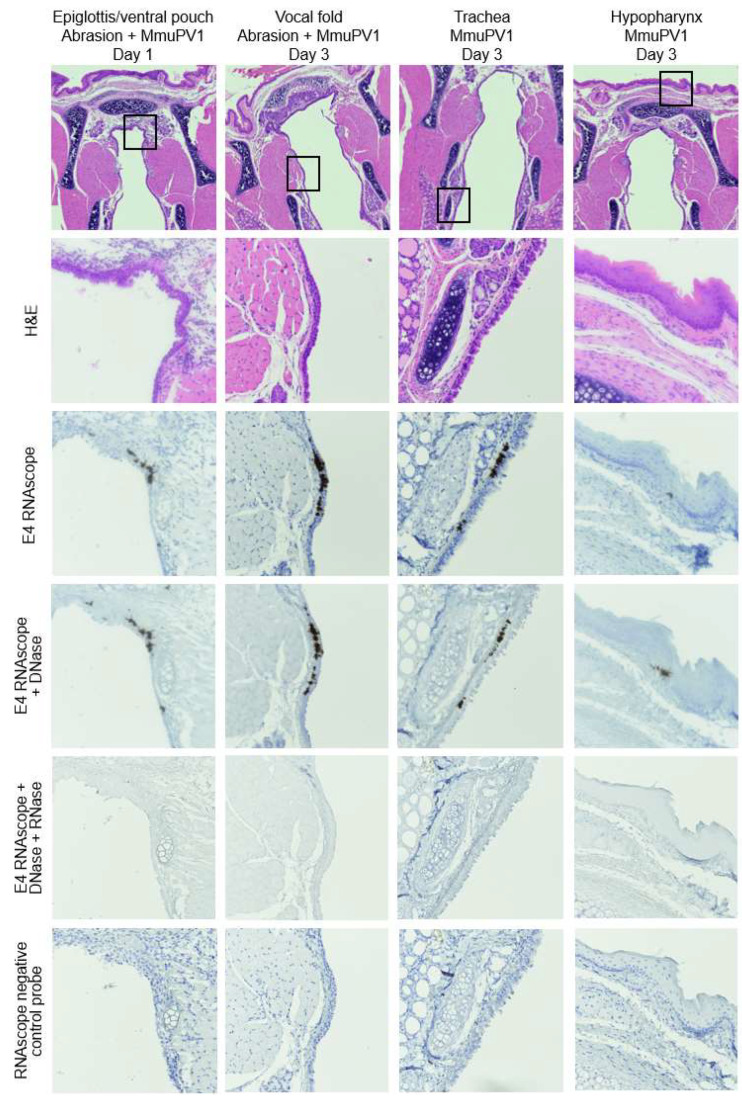
Subclinical MmuPV1 infections in larynx, trachea, and hypopharynx day 1 and 3 after infection. Low magnification of H&E-stained coronal sections of murine larynx and 40× magnifications of areas indicated in boxes with serial sections stained with H&E, MmuPV1 E4 RNAscope ISH, E4 RNAscope with DNase treatment, E4 RNAscope with DNase and RNase treatment, and negative control probe RNAscope ISH.

Subclinical laryngeal MmuPV1 infections were revealed via RNAscope at early timepoints post infection. Viral transcript was present in the ventral pouch day 1 after abrasion and infection, in vocal folds and ventral pouch (not shown) day 3 after abrasion and infection, and in trachea and hypopharynx day 3 after infection without injury (Figure 8). Signal remained after DNase treatment, but was absent after DNase and RNase treatment, confirming the presence of transcripts rather than the signal from genomic viral DNA, either inside cells or from input virus stock remaining in tissues post infection. Signal was absent with the negative control probe, indicating that results were not due to background DAB staining. RNAscope results were reviewed by the pathologist, who confirmed negative pathology. These results demonstrate that MmuPV1 can infect the larynx and produce transcripts as early as 1 day after infection. 

### 3.6. MmuPV1 RNA and Capsid Protein in Extralaryngeal Head and Neck Tissues

**Figure 9:**
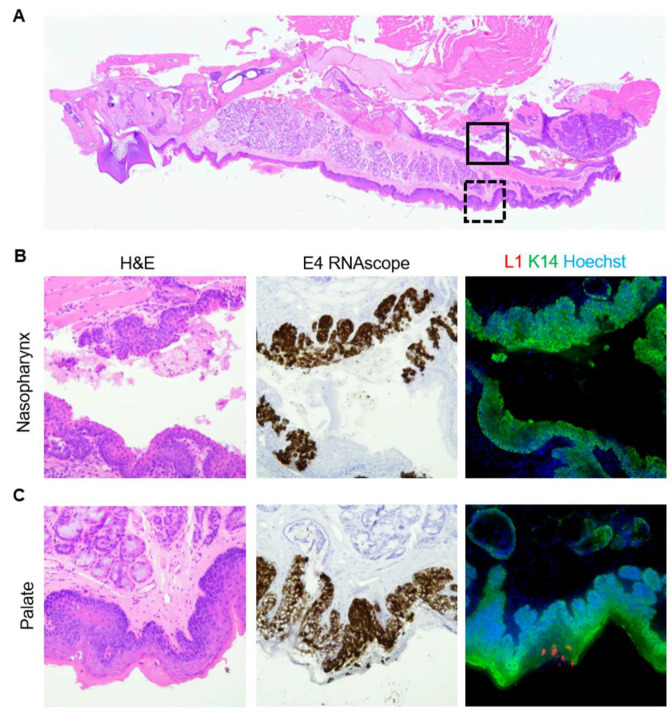
MmuPV1 capsid was produced in the infected palate, but not in the nasopharynx. (**A**) Low magnification of an H&E-stained sagittal section of a murine palate. Boxes indicate areas magnified in (**B**,**C**). (**B**) 40× magnification of solid box in (**A**). Serial sections of mild sinonasal dysplasia stained with H&E, MmuPV1 E4 ISH, and MmuPV1 L1/K14 IF. (**C**) 40× magnification of dashed box in (**A**). Serial sections of mild palate dysplasia stained with H&E, MmuPV1 E4 ISH, and MmuPV1 L1/K14 IF.

All hypopharynx disease was positive for both MmuPV1 E4 transcript and L1 capsid protein (Figure S9A,B). As previously shown [54,55], tongue and palate dysplasias were positive for transcript and capsid protein (Figure S9C–F). In the palate, only diseased stratified squamous epithelium on the oropharyngeal side was positive for L1. As in the larynx, the diseased respiratory epithelium of the nasopharyngeal side was positive for transcript, but negative for capsid protein (Figure 9).

### 3.7. MmuPV1 Spread to Lower Airways and Esophagus

**Figure 10:**
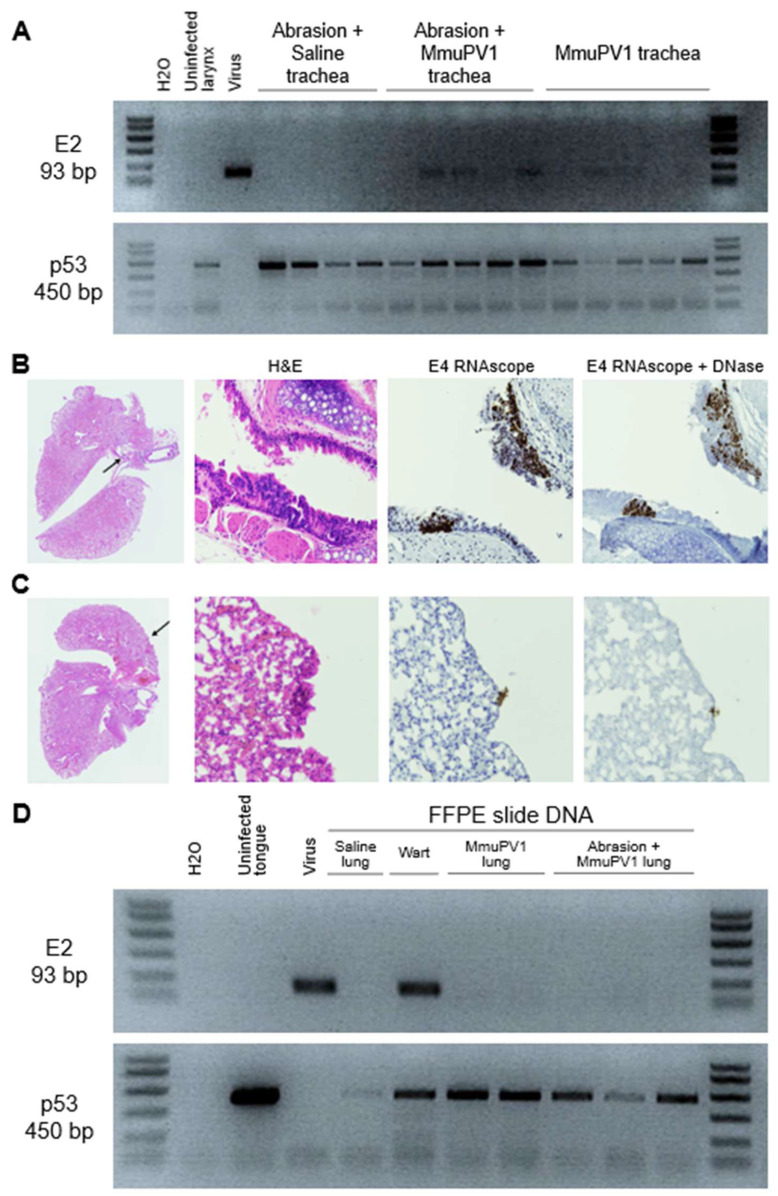
MmuPV1 infection spread to the distal trachea and bronchi, but not to the lung. (**A**) Gel electrophoresis of the PCR results for MmuPV1 E2 and *Mus musculus* p53 in distal tracheal tissue DNA at week 12 post procedure. (**B**,**C**) Low magnification of an H&E-stained section of murine lungs and 40× magnification of areas indicated with arrows, with serial sections stained with H&E, MmuPV1 E4 RNAscope ISH, and E4 RNAscope with DNase treatment. (**B**) Bronchus positive for E4. (**C**) Lung with questionable E4 signal. (**D**) Gel electrophoresis of the PCR results for MmuPV1 E2 and *Mus musculus* p53 in DNA isolated from 2 slides/4 sections of 5 um coronal lung.

We assessed the spread of MmuPV1 to the respiratory and gastrointestinal tracts because the larynx sits at the junction of these two organ systems. E2 PCR analysis of DNA isolated from distal trachea and lung tissues revealed MmuPV1 DNA in distal tracheas from infected mice at week 12 (Figure 10A). Distal tracheas harvested at earlier timepoints and all lungs were negative for MmuPV1 DNA by tissue PCR. The pathology assessment revealed atypia, or atypical cells, below the diagnostic threshold for dysplasia, in distal tracheas and bronchi collected weeks 8 and 12 from MmuPV1-infected mice with and without vocal fold abrasion. All lung pathology was negative. Bronchial atypia was positive for E4 transcript via RNAscope ISH (Figure 10B). There was one possible transcript-positive focus in the lung tissue (Figure 10C). Lung DNA was isolated from FFPE slides from all mice with tracheal or bronchial atypia, including sections serial to the suspected viral transcript-positive slide. Slides were scraped carefully to avoid collecting tracheal and bronchial tissues. PCR revealed that the lung tissue was negative for MmuPV1 DNA (Figure 10D). Taken together, these results show that laryngeal MmuPV1 infection can spread to bronchi, but not lungs, in a 12-week time period.

No esophagus tissues assessed via PCR were positive for MmuPV1 DNA. The pathology assessment revealed esophagi with mild dysplasia at weeks 2, 4, and 12 post infection. Lesions were positive for E4 transcript and L1 capsid protein (Figure S10). Diseased esophagi were few, and it is likely that the tissue sections analyzed via PCR did not contain diseased areas. 

### 3.8. Infectivity of Laryngeal Lavage and Secondary Laryngeal Infections in Nude Mice

**Figure 11:**
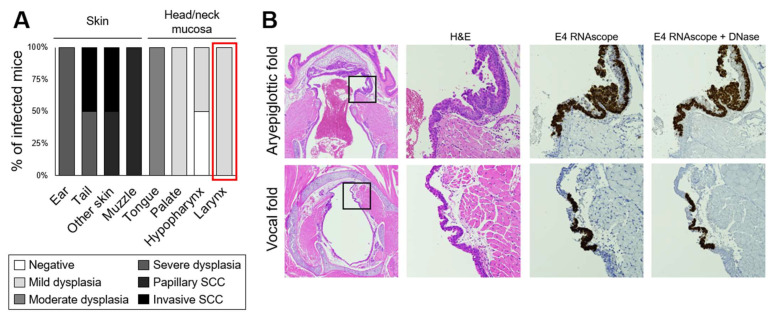
Secondary laryngeal MmuPV1 infections and dysplasias in nude mice arose after skin infection with laryngeal lavage. (**A**) Disease severity in nude mice infected with laryngeal lavage. SCC: squamous cell carcinoma. (**B**) Low magnification of H&E-stained sections of murine larynges and 40× magnification of areas indicated with boxes, with serial sections stained with H&E, MmuPV1 E4 RNAscope ISH, and E4 RNAscope with DNase treatment.

The laryngeal lavage pooled from MmuPV1-infected NSG mice harvested on week 8 was used to infect ears and tails of nude mice. Warts arose at infection sites 10 weeks after skin infection and increased in number and size until month 5, when animals were collected (Figure S11A). Warts were also observed at months 4 and 5 at additional cutaneous sites including muzzles, limbs, and around the eyes (Figure S11B). Nude mice infected with high-titer MmuPV1 stock grew warts by 5 weeks post infection (Figure S11A). Skin lesions were positive for MmuPV1 L1 capsid protein (Figure S11C). Mock infections produced no visible lesions in nude mouse skin. The pathology assessment determined that skin lesions ranged from severe dysplasia to papillary and invasive squamous cell carcinoma (Figure 11A). These results demonstrate that lavage from MmuPV1-infected larynx and immediately surrounding tissues contained infectious virus.

Nude mouse head and neck tissues were collected. Moderate dysplasia was diagnosed in tongues, while mild dysplasia was diagnosed in larynges, palates, and hypopharynx (Figure 11A). Larynges including vocal folds were positive for MmuPV1 E4 transcript (Figure 11B) and negative for capsid protein (not shown). These findings demonstrate secondary infections of unmanipulated larynx and vocal folds after primary skin infections, confirming that injury is unnecessary for vocal fold MmuPV1 infection in immunocompromised mice.

## 4. Discussion

This is the first report of experimental laryngeal infection with MmuPV1. We compared measures of laryngeal MmuPV1 infection and disease pathogenesis over 3 months between mice that underwent vocal fold abrasion and mice whose larynges were uninjured. The findings support our hypothesis that vocal fold epithelial injury enhances papillomavirus infection. Surprisingly, however, injury was not required for laryngeal infection with MmuPV1 or the development of severe disease in NSG mice. Secondary laryngeal infections and mild dysplasia developed in nude mice by 5 months after skin infection. Unlike our primary infections of NSG mouse larynges, which used a relatively high viral dose of 5.2 × 10^9^ VGE, the dose of the virus that ultimately reached the larynx from skin infections in nude mice could not be quantified, but it was likely quite low. Nude mice underwent no experimental manipulation of the larynx, pharynx, or oral cavity. Therefore, secondary laryngeal infections in nude mice definitively confirm that experimental injury is not required for MmuPV1-induced disease in the larynx of immunocompromised mice. 

MmuPV1 infection also resulted in disease of the hypopharynx and oropharynx. These sites were not intentionally injured or infected, but these results suggest that incidental MmuPV1 infection and perhaps pharyngeal injury could not be avoided in experimental procedures due to the relative sizes of endoscopic instruments and mouse pharynx. Unlike the pharynx, it is implausible that the larynx was injured incidentally in our study. The mouse larynx is significantly smaller than the operating sheath, and the tubing used for infections was able to be placed within the laryngeal surface of the epiglottis, but it could not fit between the vocal folds. Any unintentional experimental injury of the larynx and especially the vocal folds was therefore highly unlikely. MmuPV1-induced disease has been reported in other tissue sites without experimental injury, including oral, oropharyngeal, genital, and anal mucosa and tail and muzzle skin [47,55,57,58,59,73,74]. However, chewing and swallowing hard chow, grooming, and sexual intercourse could induce incidental epithelial injuries at these sites. There is no comparable activity that could incidentally injure the mouse larynx. Swallowed foods and liquids do not enter the larynx in mice, unlike the oral cavity and pharynx [75,76,77,78].

There was no sex difference in MmuPV1-associated laryngeal disease. Many more male than female humans are diagnosed with adult onset RRP, possibly due to differences in transmission linked to oral sex [25,66]. Sex difference in juvenile onset RRP is unclear [22]. Our results suggest that sex differences in laryngeal papillomavirus disease may be linked to behavior, rather than innate sex differences. These results should be confirmed in immunocompetent mice given sex differences in immunology and immune response to viral infections [79,80].

MmuPV1 infection spread to the trachea and bronchi, resulting in severe dysplasia in the proximal trachea and atypia in bronchi. Lungs were not infected by the 3-month endpoint. Lung disease is a rare, but severe, complication of RRP, while tracheal disease is more common. RRP spreads outside of the larynx in up to 48% of cases, and the trachea is the most common site involved [81]. Disease spreads to the lungs in only 3% of cases [82]. Unintentional tracheal injury in our model was even less likely than unintentional laryngeal injury, for the reasons discussed above. A high dose of input virus likely reached the trachea and is one possible explanation for our findings. However, the respiratory epithelium of the nasopharynx, farther from the primary infection site, was also infected and developed disease. Nasopharynx likely received a low dose of the virus given that the possible routes of infection at this site were incidental exposure to input virus during infection procedures, new MmuPV1 virions produced in hypo- or oropharynx over the course of the study, or naked MmuPV1 DNA from desquamating laryngeal or respiratory cells. Taken together, these data suggest that laryngeal and respiratory epithelia are especially vulnerable to MmuPV1 in NSG mice.

Similarities and differences between laryngeal disease induced by MmuPV1 in NSG mice and RRP are presented in Table 1. Pathology and endoscopy revealed that MmuPV1-induced laryngeal lesions were not papillomas, but relatively flat, smooth dysplasias and squamous cell carcinomas. Laryngeal dysplasia was severe by week 2, cancers developed by week 8, and all laryngeal disease was severe dysplasia or cancer at week 12. By contrast, in RRP, which can persist for decades, moderate or greater dysplasia is found in less than 20% of cases [83,84,85] and lesions undergo malignant transformation in <1% of children and 3–6% of adults [86]. The percent of laryngeal cancers that arise from RRP is unknown, but it is likely very small. A large meta-analysis found that 25% of laryngeal cancers are HPV positive [87]. Low- vs. high-risk HPV was not assessed, and these data may be imprecise because routine HPV testing is not conducted in laryngeal cancers due to unknown prognostic significance outside the oropharynx. MmuPV1 is capable of causing pathology-confirmed papillomas in skin, vagina, and tongue [88,89], but these also progress to cancer in immunocompromised mice [51,54,90]. Future studies in immunocompetent mice will explore the ability of MmuPV1 to cause benign papillomas in the larynx.

MmuPV1 E4 transcripts were produced in all diseased laryngeal epithelium, regardless of injury, and in diseased pharynx and trachea. This is consistent with past studies of MmuPV1 infection of cutaneous and mucosal epithelia [51,52,54,55,70,73]. RNAseq analysis has revealed abundant E1^E4 transcripts in RRP lesions [91]. We also detected MmuPV1 E4 transcripts in larynges without disease by day 1 after infection. Low levels of MmuPV1 E1^E4 spliced transcripts have been detected in mouse skin by real-time PCR day 4 after infection [73]. Subclinical infections in RRP patients have been reported, characterized by low-risk HPV DNA and transcripts in clinically normal laryngeal and tracheal tissues [38,39,103,104]. However, RRP patients’ disease can remain subclinical for years to decades, while all mice in our study developed disease by 3 months post infection. 

MmuPV1 L1 capsid protein was absent from the larynx at all stages of disease, except in one animal with squamous metaplasia at the level of the cricoid cartilage, likely associated with injury [72,105]. This differs from MmuPV1-induced oropharyngeal dysplasias and cancers described in this and other studies [54,55], as well as disease in the anal tract [52], female genital tract [51,59], and skin warts and cancers [70,71]. In a study of sexual MmuPV1 transmission, L1 was found less often than viral transcripts in the penises of male mice, but it was not absent [59]. In historical electron microscopy [92,93,94,95,96] and immunostaining [97,98,99,100,101,102] experiments, HPV virions were very low to absent in RRP lesions, which may have delayed the discovery of the viral etiology of the disease. More specific IHC results revealed that L1 was found in sparse and superficial RRP cells [106]. A recent transcriptomic analysis of lesions from 15 RRP patients revealed that L1 and L2 transcripts were absent from several patients’ lesions, but present in most [91]. The absence of productive HPV infection in a subset of RRP patients, and few cells producing virions in most patients, suggests that there must be another mechanism for viral persistence in the larynx besides the completion of the low-risk HPV life cycle. Our model may be particularly relevant to this subset of patients. 

Similar to the larynx, the respiratory epithelia of the trachea and nasopharynx also developed disease and expressed MmuPV1 E4 transcript, but not L1 capsid protein. Therefore, both the laryngeal and respiratory epithelia of immunocompromised mice are susceptible to MmuPV1-induced disease in the absence of productive infection. Of note, laryngeal lavage was able to infect nude mouse skin and produce warts. The source of infectious material could have been virions produced in the epithelium of the hypopharynx, which was also lavaged. Another possibility is that desquamated laryngeal or tracheal cells in lavage contained naked MmuPV1 DNA, which is infectious on its own without being encapsidated [73].

Hypotheses for differences between laryngeal disease induced by MmuPV1 and RRP include differences in host species, viruses, and immunology. It is well known that papillomaviruses require stratified squamous epithelium for productive infection. Thus, unlike in humans, vocal folds and other stratified squamous epithelium inside the mouse larynx may be unable to support productive MmuPV1 infection. This inability may be due to insufficient cell layers, since murine vocal fold epithelium has only 2–4 layers compared to 5–10 in humans [31,107]. Alternatively, murine vocal fold epithelium may more closely resemble intermediate epithelium than human vocal fold epithelium does on a continuum between stratified squamous and respiratory. For example, the vocal fold epithelium in both species expresses cytokeratin 13 (K13) in differentiated cells, while the tracheal epithelium does not [108,109]. However, the apical layers of the vocal fold epithelium are cytokeratin K8+ in mice but K8- in humans, while laryngeal sites that contain both intermediate and respiratory epithelium, as well as the trachea, are K8+ in both species [107,108,109,110,111]. 

Differences between high- and low-risk HPVs and MmuPV1 may have contributed to our results. MmuPV1 was discovered as a cutaneous virus [47], but has been used to model mucosal cancers associated with high-risk HPVs in the head and neck [53,54,55], genital tract [51], and anus [52]. However, MmuPV1 binds different components of the basement membrane during entry into basal cells than high- or low-risk HPVs [112]. MmuPV1 differs from high-risk HPVs in the absence of an E5 open reading frame [70], transcription of E6 and E7 oncogenes from different promoters [70], and specific mechanisms of the E6 and E7 proteins’ contribution to oncogenesis [113,114]. As in high-risk HPVs, low-risk HPVs that cause RRP do have an E5 gene [91], but unlike high-risk HPVs and as in MmuPV1, E6 and E7 are transcribed from separate promoters [115]. Low-risk HPV E5, E6, and E7 proteins have weaker or altered functions compared to their high-risk counterparts [115]. As in high-risk HPVs, MmuPV1 can integrate into the host genome in diseased tissues [116]. Low-risk HPV genomes generally do not integrate in RRP [91]. It is not known why MmuPV1 caused dysplasias and cancers rather than papillomas in our experiments. Potential explanations that can be tested empirically are the lack of an E5 gene in MmuPV1 and the lack of an immune response to the virus in NSG mice.

The mice in this foundational study of laryngeal MmuPV1 infection were severely immunocompromised. NSG mice are deficient in B cells, T cells, and functional natural killer (NK) cells [64], and nude mice lack functional T cells [117]. In contrast, most RRP patients are immunocompetent and mount normal immune responses to other pathogens [118]. This limits the generalization of our findings to human disease. The lack of an immune response to MmuPV1 could have resulted in the severe disease phenotypes observed in our study. We hypothesize that extending our work to immunocompetent mice will produce benign disease phenotypes, since MmuPV1 is capable of causing benign papillomas in addition to cancers [48,49,50]. Hypotheses for tissue-specific mechanisms underlying the laryngeal vulnerability to papillomaviruses warrant further study in immunocompetent mice. We expect some immunocompetent mice to clear the virus quickly and some to develop persistent disease, as shown in other tissues [48,49,50,119]. Once we identify immunocompetent mice that can sustain laryngeal disease induced by MmuPV1, tissue-specific mechanisms can be explored. An immunocompetent animal model would then allow studies of latency and recurrence, key features of RRP. Long-term latent rabbit oral papillomavirus infection and reactivation has been experimentally produced in rabbit tongue with the manipulation of immunosuppression [120,121]. MmuPV1 skin warts regress with the withdrawal of immunosuppression [122]. Similar studies in the mouse larynx will be invaluable next steps in developing this novel preclinical animal model of RRP. 

## 5. Conclusions

MmuPV1 laryngeal infection caused disease in 100% of infected mice by 3 months post infection. Unlike in other experimentally infected tissues, injury was absolutely dispensable for infection and disease pathogenesis in immunocompromised mice. This apparently unique vulnerability of the larynx to papillomavirus disease will be further explored in future work. Similar to RRP, MmuPV1 transcript was detected in all infected laryngeal tissues as well as clinically normal tissue with subclinical infection. Productive infection arose in laryngeal tissue in which injury produced squamous metaplasia. MmuPV1 spread to the trachea and bronchi and induced disease. Unlike RRP, MmuPV1-induced laryngeal lesions were relatively flat dysplasias that progressed to cancer, rather than exophytic, clustered, stippled papillomas. MmuPV1 capsid protein was absent from the typical infected mouse larynx, while it is very sparse in RRP tissues but not absent. All animal papillomaviruses provide imperfect, but useful, models for human disease. As with other diseases modeled with MmuPV1, the degree of experimental manipulation of both the virus and host that is now possible in a laboratory mouse model can provide important insights into RRP despite differences between mouse and human laryngeal papillomavirus infections. Therefore, this first report of laryngeal MmuPV1 infection provides a foundation for a preclinical model of RRP. Our companion report (forthcoming) describes laryngeal epithelial changes in response to MmuPV1 infection.

## Figures and Tables

**Table 1:** Disease similarities and differences between vocal fold disease in NSG mice infected with MmuPV1 and RRP.

Disease Feature	NSG Mouse Vocal Fold + MmuPV1	RRP
Benign disease	Yes	Yes [1,2,3]
Moderate or higher dysplasia	Ubiquitous by 3 months	Rare [83,84,85]
Progression to cancer	Frequent	Extremely rare [86]
Spread to trachea and bronchi	Yes	Yes [81]
Spread to lung	No	Extremely rare [82]
Viral gene expression	Yes	Yes [91]
Capsid production	Squamous metaplasia only	Sparse, absent in some patients [91,92,93,94,95,96,97,98,99,100,101,102]

## Data Availability

All relevant data are provided within the article and Supplementary Materials.

## Supplementary Materials

The following supporting information can be downloaded at: https://www.mdpi.com/article/10.3390/v14051000/s1, Table S1: Type 3 tests of fixed effects on weight as percentage of baseline; Figure S1: Weight change over time differed by treatment group; Table S2: Reduced weight gain in mice with a laryngeal MmuPV1 infection; Figure S2: Oral swab PCR; Figure S3: Laryngeal lavage PCR; Figure S4: Gross and microscopic epiglottic lesions; Figure S5: MmuPV1-induced disease differed among laryngeal tissues; Figure S6: MmuPV1-induced laryngeal disease did not differ between males and females; Figure S7: MmuPV1 DNA was absent from FFPE larynx slides of mock-infected mice with laryngeal dysplasia; Figure S8: MmuPV1 E4 and L1 transcripts colocalized in the larynx and hypopharynx; Figure S9: MmuPV1-induced hypopharynx, tongue, and palate dysplasia was positive for E4 transcript and L1 capsid protein; Figure S10: MmuPV1 infection spread to the esophagus; Figure S11: Laryngeal lavage from MmuPV1-infected NSG mice produced skin cancers in nude mice.
