# Supplementary material for: A Novel In Vivo Model of Laryngeal Papillomavirus-Associated Disease Using Mus musculus Papillomavirus
Source: Viruses. 2022 May 8;14(5):1000. doi: 10.3390/v14051000 (PMC9147793; doi:10.3390/v14051000)
Supplement: Supplementary file 1 [file viruses-14-01000-s001.zip › viruses-1693716-supplementary.pdf]

Supplementary Materials

A Novel In Vivo Model of Laryngeal Papillomavirus-Associated Disease Using *Mus musculus* Papillomavirus

Renee E. King [1,2], Andrea Bilger [1], Josef Rademacher [2], Ella T. Ward-Shaw [1], Rong Hu [3], Paul F. Lambert [1] and Susan L. Thibeault [2,*]

[1] McArdle Laboratory for Cancer Research, Department of Oncology, University of Wisconsin-Madison, Madison, WI 53705, USA; renee.king@wisc.edu (R.E.K.); bilger@oncology.wisc.edu (A.B.); etward@wisc.edu (E.T.W.-S.); plambert@wisc.edu (P.F.L.)

[2] Department of Surgery, University of Wisconsin-Madison, Madison, WI 53705, USA; rademacher3@wisc.edu (J.R.)

[3] Department of Pathology and Laboratory Medicine, University of Wisconsin-Madison, Madison, WI 53705, USA; rhu6@wisc.edu (R.H.)

* Correspondence: thibeault@surgery.wisc.edu (S.L.T.)

Table S1. Type 3 tests of fixed effects on weight as percentage of baseline.

Effect	df	F	*p*	Interpretation
Group	3916	7.48	<0.0001	Deferred to simple effects within interaction effects
Time	14,916	11.20	<0.0001	Deferred to simple effects within interaction effects
Sex	1916	5.31	0.0214	Deferred to simple effects within interaction effects
Age	1916	0.01	0.9068	No independent effect of baseline age on % weight change
Group × Time	40,916	2.46	<0.0001	Group differences in % weight change over time
Group × Sex	3916	6.09	0.0004	Group differences in average % weight change across timepoints by sex
Sex × Time	14,916	5.91	<0.0001	Sex differences in % weight change over time

Weight change as a percentage of baseline weight was tested in linear mixed models with treatment group (Abrasion + MmuPV1, MmuPV1, Abrasion + Saline, Saline), week post procedure, sex, and interactions of these variables as fixed effects and animal ID as a repeated effect. Age in days was also included to test for an independent effect on weight change since weight gain is generally faster in younger mice. Control animals that did not undergo laryngeal or oral procedures were not included because they were collected at week 2 and there were no data for this group at later timepoints. Group × Time × Sex 3-way interaction was not significant and was removed from the model. df: degrees of freedom. F: F statistic.

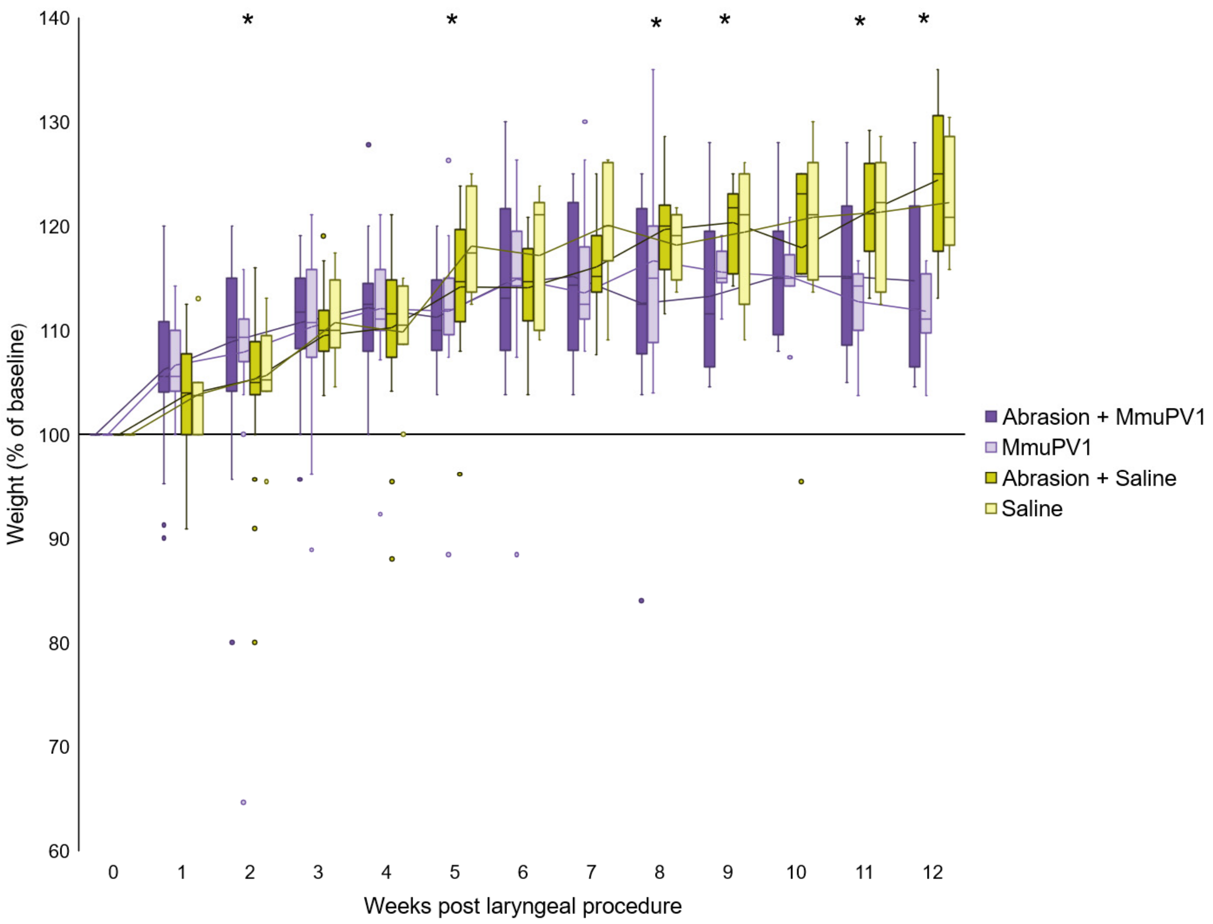

Figure S1. Weight change over time differed by treatment group. Group differences at each timepoint within significant Group × Time interaction in mixed model. Day 1 and 3 data not shown. *Tukey-adjusted $p < .05$ for group difference at a given timepoint.

Table S2. Reduced weight gain in mice with laryngeal MmuPV1 infection.

**Week**	**Significant pairwise differences**	**β ± SE**	**Adj. *p***
2	Abrasion + MmuPV1 > Abrasion + Saline	4.0% ± 1.3%	0.0160
5	Abrasion + MmuPV1 < Saline	-6.7% ± 2.5%	0.0379
8	Abrasion + MmuPV1 < Abrasion + Saline	-7.3% ± 2.0%	0.0016
9	Abrasion + MmuPV1 < Abrasion + Saline	-7.3% ± 2.8%	0.0414
11	MmuPV1 < Abrasion + Saline	-8.7% ± 2.9%	0.0142
	MmuPV1 < Saline	-8.8% ± 2.8%	0.0090
12	Abrasion + MmuPV1 < Abrasion + Saline	-9.6% ± 2.9%	0.0053
	Abrasion + MmuPV1 < Saline	-7.8% ± 2.8%	0.0284
	MmuPV1 < Abrasion + Saline	-12.6% ± 2.9%	<0.0001
	MmuPV1 < Saline	-10.9% ± 2.8%	0.0006

Significant pairwise differences and p-values for weight change among treatment groups after Tukey adjustment for multiple comparisons. β: estimate. SE: standard error.

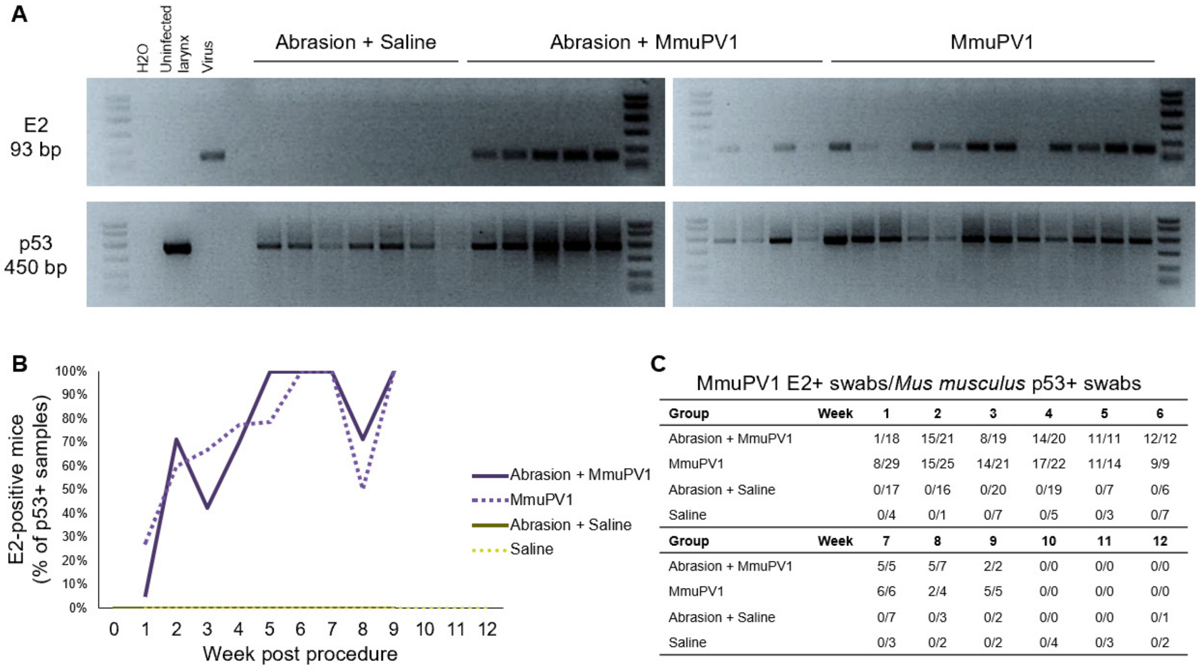

MmuPV1 E2+ swabs/*Mus musculus* p53+ swabs

Group	Week	1	2	3	4	5	6
Abrasion + MmuPV1		1/18	15/21	8/19	14/20	11/11	12/12
MmuPV1		8/29	15/25	14/21	17/22	11/14	9/9
Abrasion + Saline		0/17	0/16	0/20	0/19	0/7	0/6
Saline		0/4	0/1	0/7	0/5	0/3	0/7
**Group**	**Week**	**7**	**8**	**9**	**10**	**11**	**12**
Abrasion + MmuPV1		5/5	5/7	2/2	0/0	0/0	0/0
MmuPV1		6/6	2/4	5/5	0/0	0/0	0/0
Abrasion + Saline		0/7	0/3	0/2	0/0	0/0	0/1
Saline		0/3	0/2	0/2	0/4	0/3	0/2

Figure S2. Oral swab PCR. **(a)** Gel electrophoresis of PCR results for MmuPV1 E2 and *Mus musculus* p53 in swab DNA from 6 cages of mice at week 2 post procedure. **(b)** Line graph of E2-positive samples as a percentage of samples positive for control gene p53. **(c)** Number of p53-positive swabs that were also E2-positive.

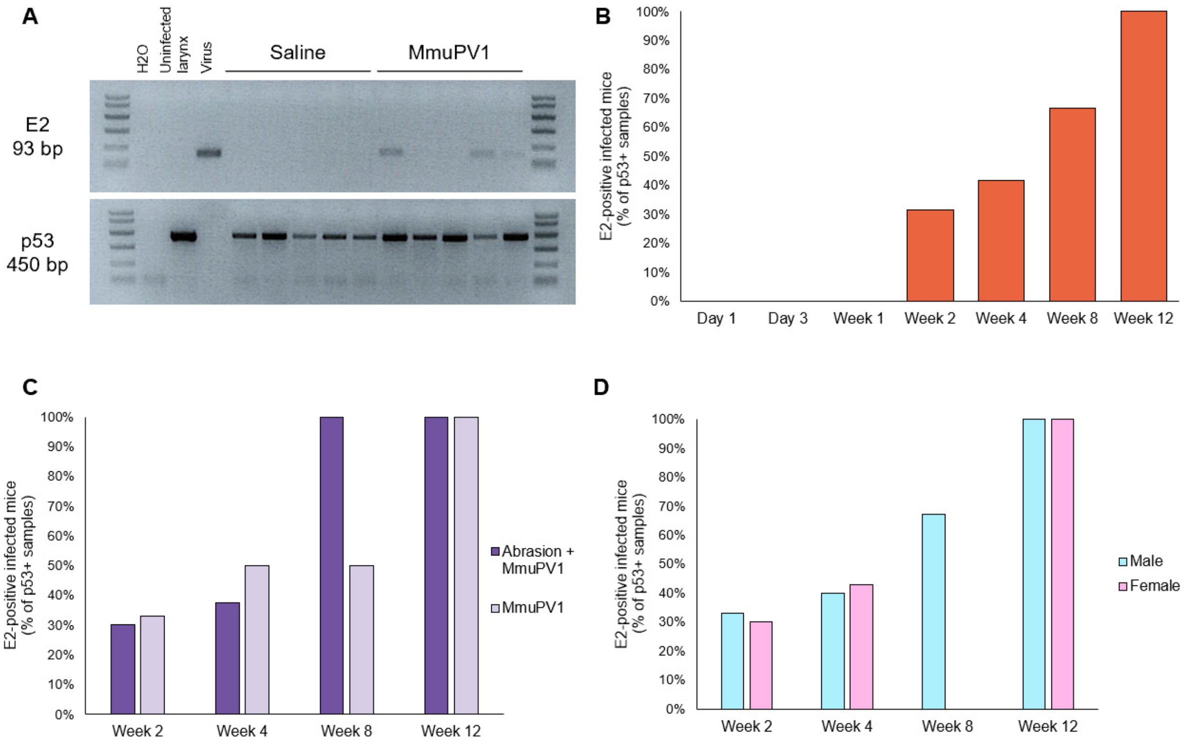

Figure S3. Laryngeal lavage PCR. **(a)** Gel electrophoresis of PCR results for MmuPV1 E2 and *Mus musculus* p53 in swab DNA from 2 cages of mice at week 2 post procedure. **(b)** E2-positive lavage samples from infected mice as a percentage of samples positive for control gene p53. Total p53-positive samples: day 1 n = 5, day 3 n = 4, week 1 n = 16, week 2 n = 19, week 4 n = 12, week 8 n = 3, week 12 n = 12. **(c)** E2-positive lavage samples with vocal fold abrasion (p53-postive samples: week 2 n = 10, week 4 n = 8, week 8 n = 2, week 12 n = 6) and without vocal fold abrasion (p53-postive samples: week 2 n = 9, week 4 n = 4, week 8 n = 1, week 12 n = 6). Fisher's exact tests: all p > .05. **(d)** E2-positive samples in males (p53-positive samples: week 2 n = 9, week 4 n = 5, week 8 n = 3, week 12 n = 6) and females (p53-positive samples: week 2 n = 10, week 4 n = 7, week 8 n = 0, week 12 n = 6). Fisher's exact tests: all p > .05.

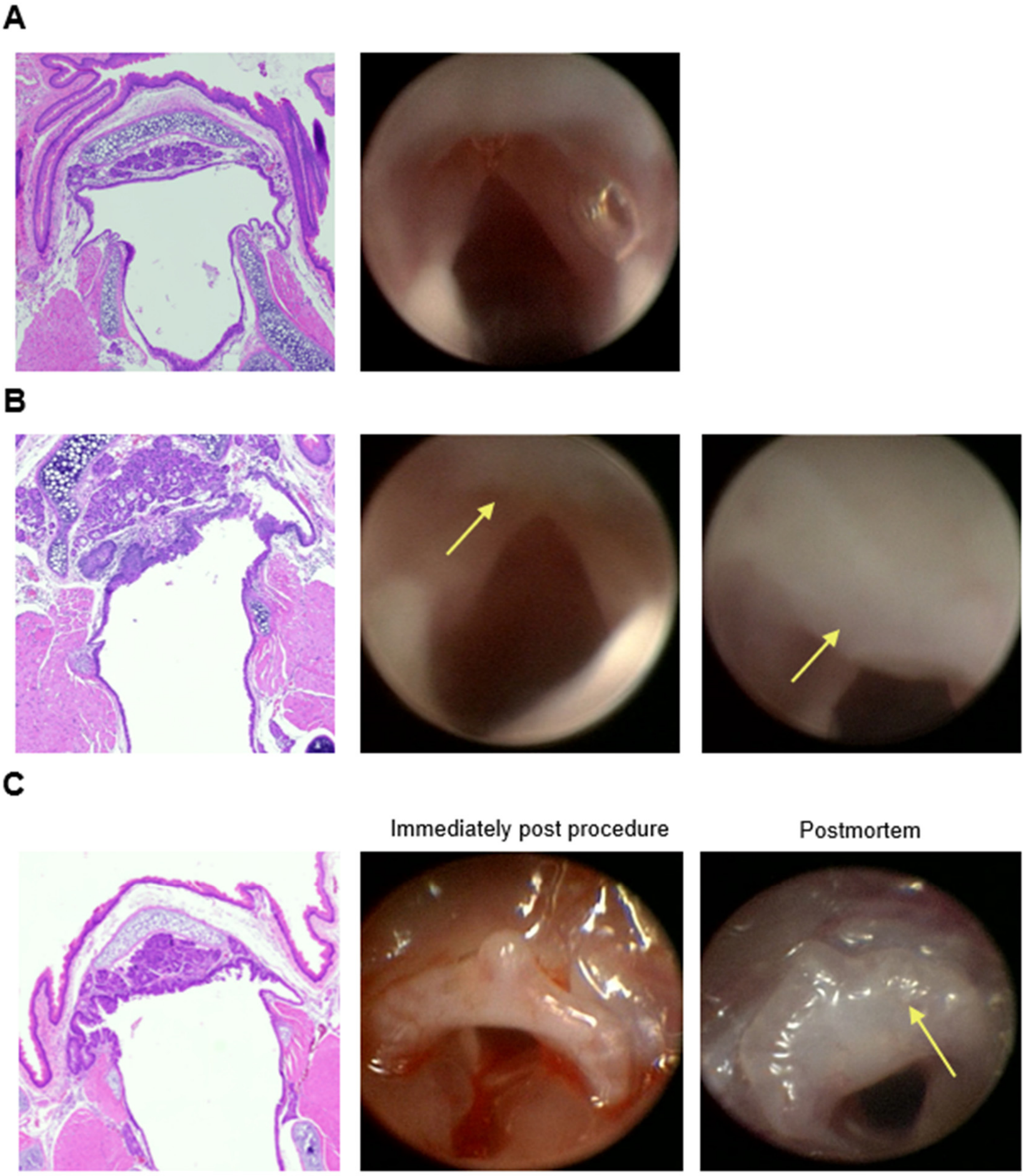

Figure S4. Gross and microscopic epiglottic lesions. H&E-stained histologic and endoscopic images of laryngeal epiglottis from the same animal. **(a)** Week 12 post saline mock infection. A bubble of secretions is visible on the right side of the image. **(b)** Week 4 post MmuPV1 infection. Arrows indicate thickened epiglottic epithelium. **(c)** Week 12 post abrasion and MmuPV1 infection. Arrow indicates bumpy epiglottis epithelium on postmortem endoscopy, in contrast to smooth epithelium in the same location on the procedure date.

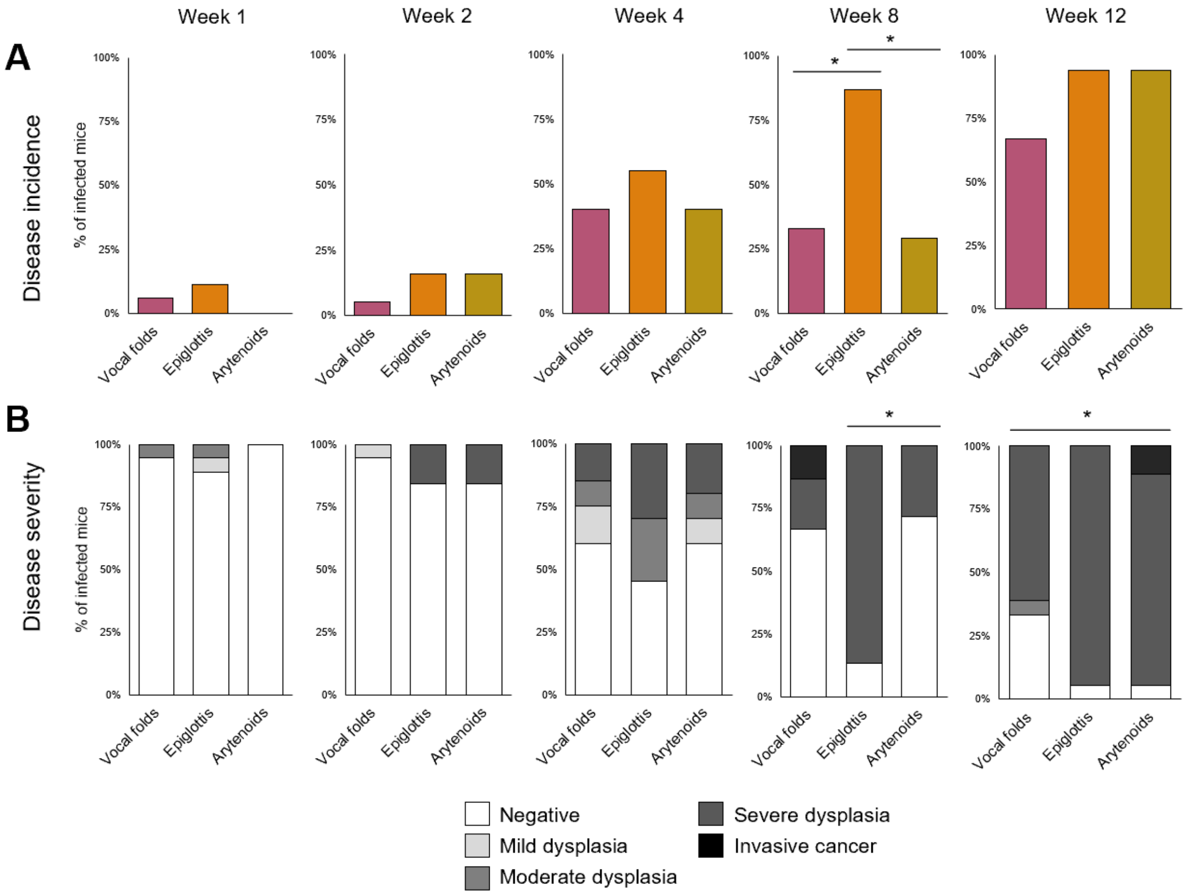

Figure S5. MmuPV1-induced disease differed among laryngeal tissues. Infected groups week 1 and later only, n = 15-20 mice per timepoint. **(a)** Disease incidence. Fisher's Exact tests of differences in dysplasia incidence among laryngeal tissues at each timepoint: week 8 $p = .0028$, week 12 $p = .0372$. All other $p > .05$. *Significant post hoc Bonferroni-adjusted Fisher's Exact pairwise tests among laryngeal tissues: week 8 vocal folds vs. epiglottis $p = .0078$, arytenoids vs. epiglottis $p = .0025$. All other pairwise tests $p > .05 / 3 = .0167$. **(b)** Disease severity. Severity coded ordinally: negative = 0, mild dysplasia = 1, moderate dysplasia = 2, severe dysplasia = 3, invasive cancer = 4. Kruskal-Wallis tests of severity among laryngeal tissues at each timepoint: week 8 $p = .0087$, week 12 $p = .0068$. All other $p > .05$. *Significant post hoc DSCF pairwise tests among laryngeal tissues: week 8 epiglottis vs. arytenoids $p = .0052$; week 12 vocal folds vs. arytenoids $p = .0253$. All other pairwise tests: adjusted $p > .05$.

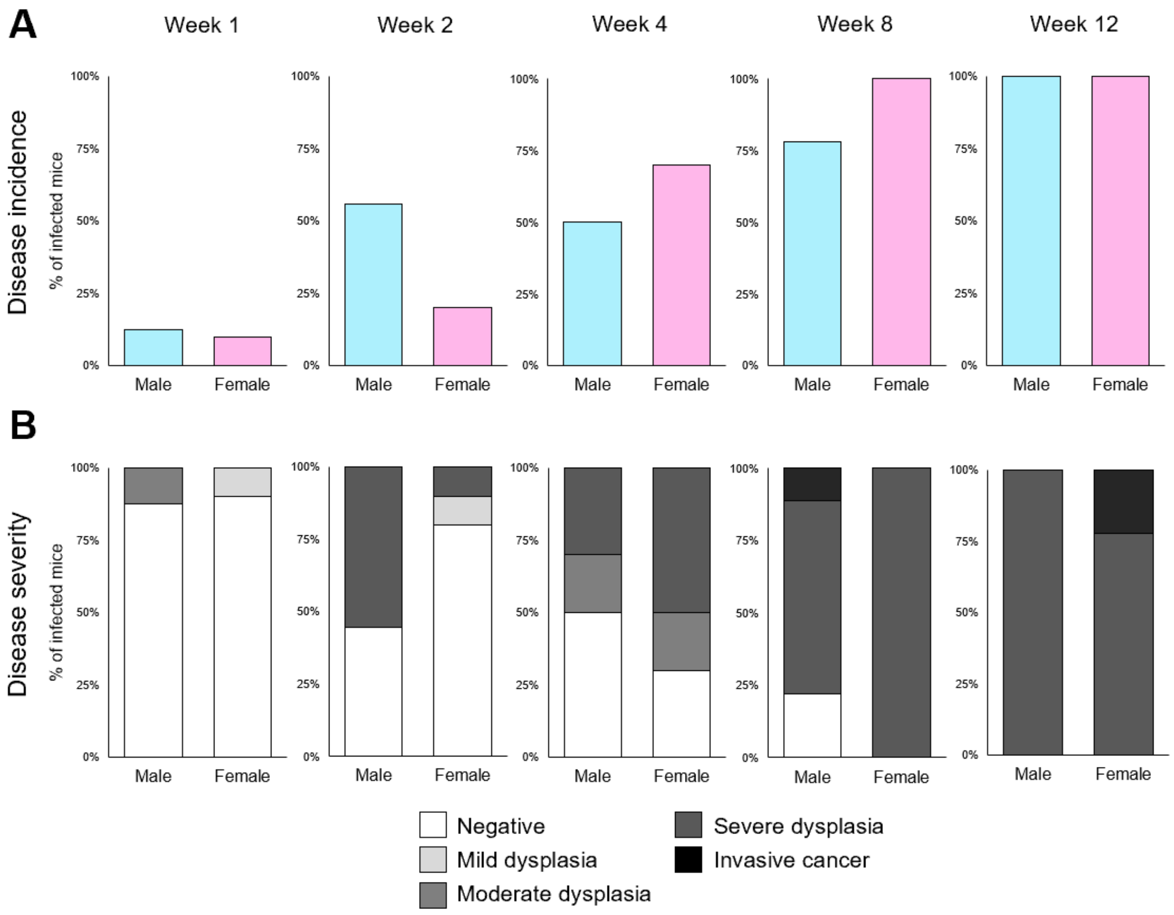

Figure S6. MmuPV1-induced laryngeal disease did not differ between males and females. Infected groups week 1 and later only, n = 7-10 mice per group per timepoint. **(a)** Disease incidence. Fisher's Exact tests of sex difference in dysplasia incidence in the larynx at each timepoint: all $p > .05$. **(b)** Disease severity. Severity coded ordinally: negative = 0, mild dysplasia = 1, moderate dysplasia = 2, severe dysplasia = 3, invasive cancer = 4. Two-tailed Wilcoxon rank-sum tests of severity in males vs. females at each timepoint: all $p > .05$.

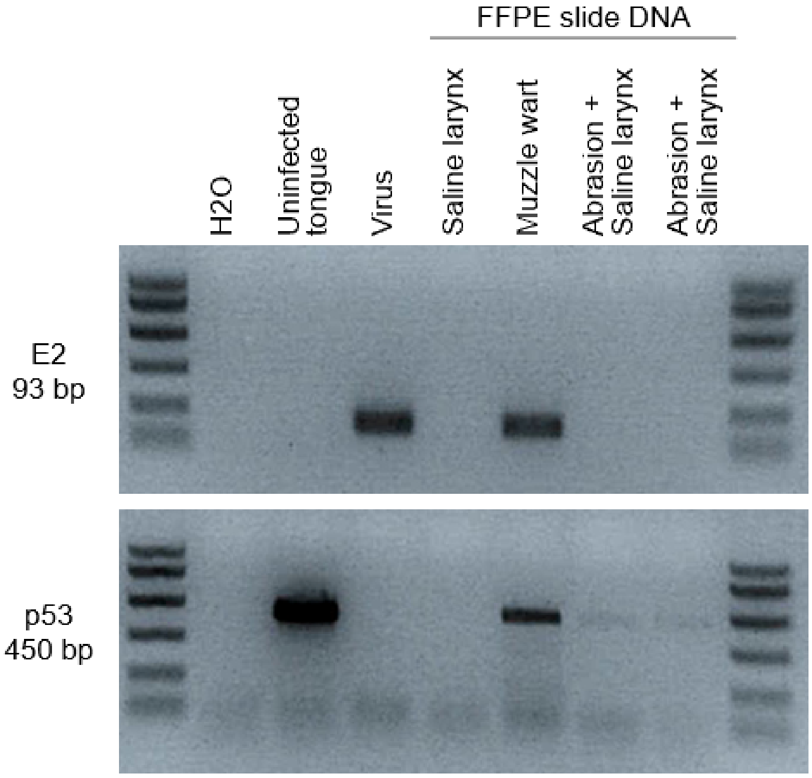

Figure S7. MmuPV1 DNA was absent from FFPE larynx slides of mock-infected mice with laryngeal dysplasia. Gel electrophoresis of PCR results for MmuPV1 E2 and *Mus musculus* p53 in DNA isolated from 2 slides/4 sections of 5 um coronal larynx. DNA from 2 slides of a muzzle wart induced by MmuPV1 was used as an additional positive control.

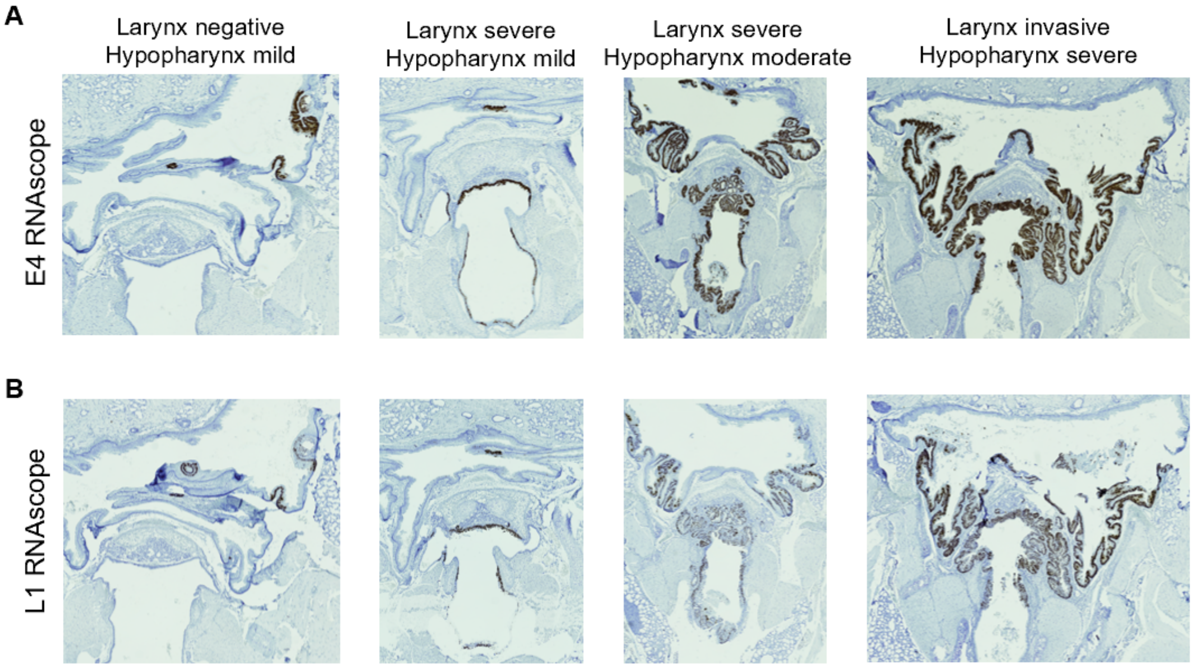

Figure S8. MmuPV1 E4 and L1 transcripts colocalized in the larynx and hypopharynx. Low magnifications of coronal murine laryngeal sections. **(a)** E4 RNAscope in varying degrees of larynx and hypopharynx disease. **(b)** L1 RNAscope in varying degrees of larynx and hypopharynx disease.

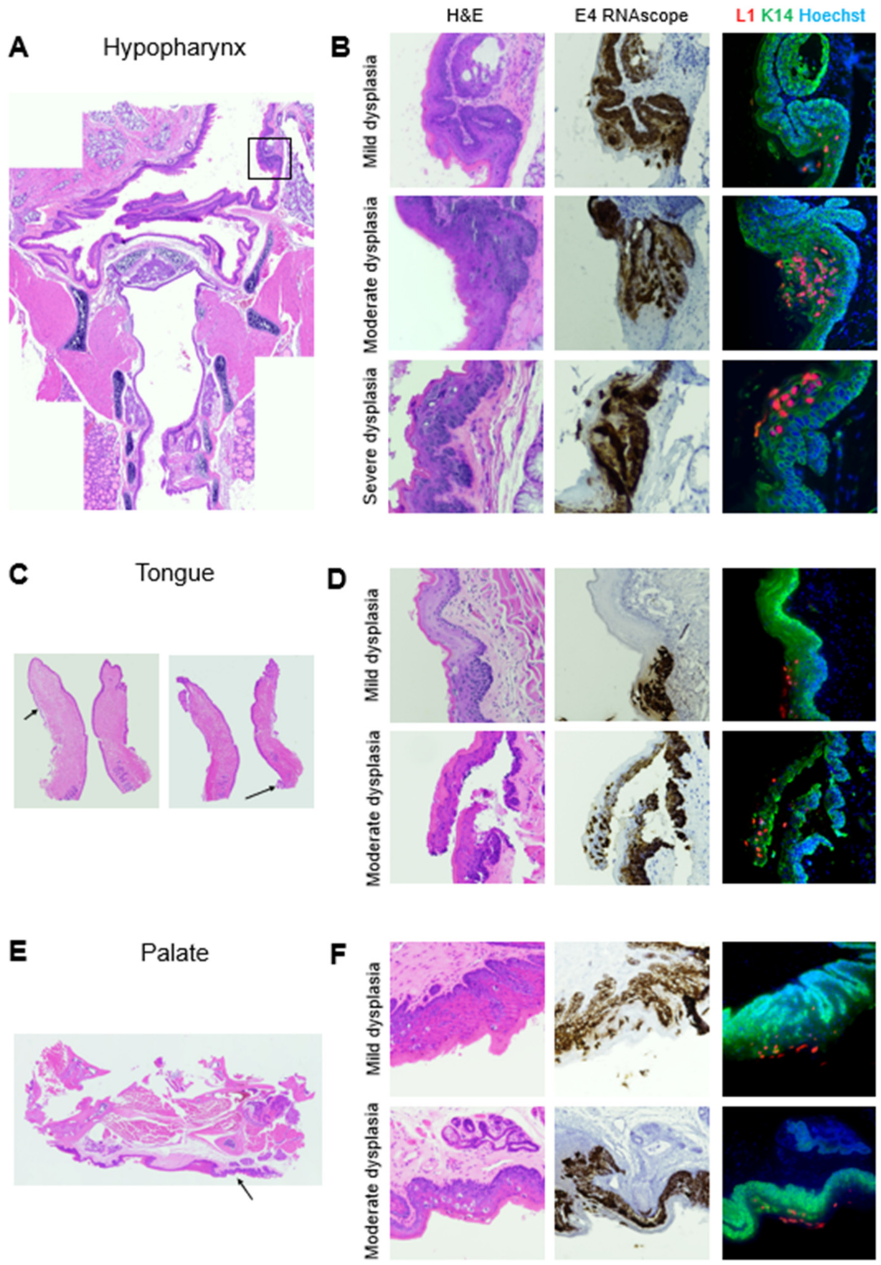

Figure S9. MmuPV1-induced hypopharynx, tongue, and palate dysplasia was positive for E4 transcript and L1 capsid protein. **(a)**, **(c)**, and **(e)** Low magnification of H&E-stained tissues. **(a)** Coronal section of murine larynx and hypopharynx. Box indicates area magnified in (b). **(c)** and **(e)** Sagittal sections of murine tongue **(c)** and palate **(e)**. Arrows indicate areas magnified in (d) and (f). **(b)**, **(d)**, and **(f)** Serial sections of mild, moderate, and severe hypopharynx dysplasia **(b)** and mild and moderate tongue **(d)**, and palate **(f)** dysplasia stained with H&E, MmuPV1 E4 ISH, and MmuPV1 L1/K14 IF. 40X.

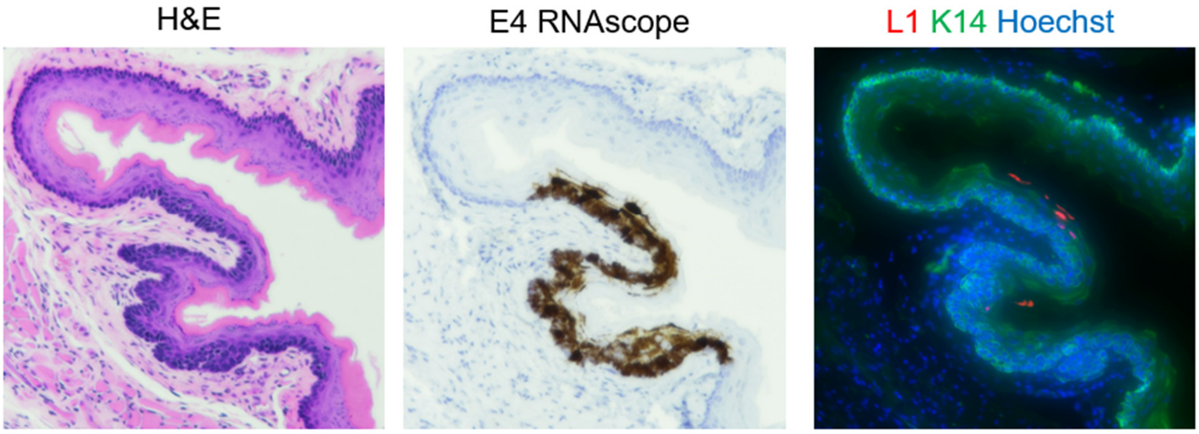

Figure S10. MmuPV1 infection spread to the esophagus. Serial sections of mild epiglottis dysplasia stained with H&E, MmuPV1 E4 ISH, and MmuPV1 L1/K14 IF. 40X magnification.

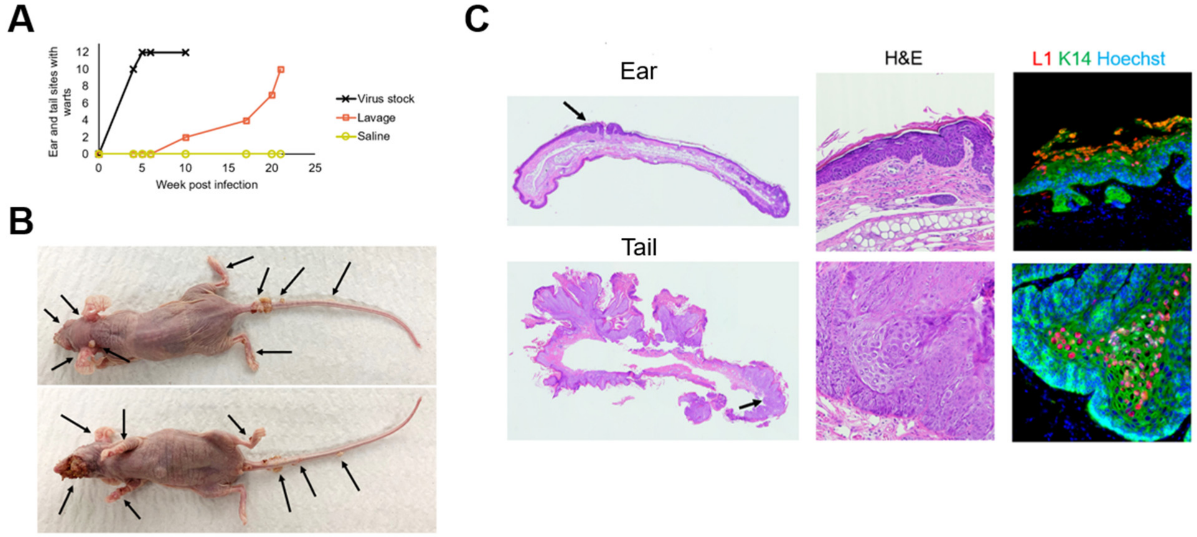

Figure S11. Laryngeal lavage from MmuPV1-infected NSG mice produced skin lesions in nude mice. **(a)** Number of ear and tail sites with warts over time in nude mice infected with high-titer MmuPV1 stock, laryngeal lavage, and mock-infected with saline. Mice infected with high-titer virus stock were harvested at week 10 for a new virus prep. **(b)** Dorsal and ventral view of nude mouse skin 5 months after skin infections with lavage. Arrows indicate warts. **(c)** Low magnification of H&E-stained sections of severe dysplasia in ear and papillary squamous cell carcinoma in tail and 40X magnification of areas indicated with arrows, with serial sections stained with H&E and MmuPV1 L1/K14 IF.
